# Data on diverse roles of helix perturbations in membrane proteins

**DOI:** 10.1016/j.dib.2016.10.023

**Published:** 2016-11-01

**Authors:** Ashish Shelar, Manju Bansal

**Affiliations:** Molecular Biophysics Unit, Indian Institute of Science, Bangalore 560012, Karnataka, India

**Keywords:** Membrane proteins, Helix kink, Helix interactions

## Abstract

The various structural variations observed in TM helices of membrane proteins have been deconstructed into 9 distinct types of helix perturbations. These perturbations are defined by the deviation of TM helices from the predominantly observed linear α-helical conformation, to form 3_10_- and π-helices, as well as adopting curved and kinked geometries. The data presented here supplements the article ‘Helix perturbations in Membrane Proteins Assist in Inter-helical Interactions and Optimal Helix Positioning in the Bilayer’ (A. Shelar, M. Bansal, 2016) [1]. This data provides strong evidence for the role of various helix perturbations in influencing backbone torsion angles of helices, mediating inter-helical interactions, oligomer formation and accommodation of hydrophobic residues within the bilayer. The methodology used for creation of various datasets of membrane protein families (Sodium/Calcium exchanger and Heme Copper Oxidase) has also been mentioned.

**Specifications Table**

**Table:** 

Subject area	*Biology*
More specific subject area	*Membrane protein structure and folding, Bioinformatics*
Type of data	*Tables and figures*
How data was acquired	*Data was retrieved from public databases*
Data format	*Analyzed data*
Experimental factors	*Protein structures were retrieved from OPM database and analyzed. Sequence and structural alignments of proteins were performed using Clustal Ω and MAPSCI respectively*
Experimental features	*This work uses X-ray crystal structure data of membrane proteins that has been deposited in the Protein Data Bank (PDB)*
Data source location	*Bangalore, India*
Data accessibility	*Data is within this article. Membrane protein structures aligned along the Z-axis can be readily retrieved from the OPM database* (http://opm.phar.umich.edu/download.php).

**Value of the data**

• The data on different types of helices shows that, apart from the commonly observed α-helices, 3_10_ and π-helices are also present within the bilayer and have varying lengths as well as distinct sequence signatures. This data provides experimentalists with options to model new 3_10_- and π-helices in the bilayer and reorient the locations of active sites in TM helices.
• The data on backbone torsion angle variation in perturbed helices indicates that in these regions the disrupted hydrogen bonds lead to free NH– and C=O groups that mediate inter-helical interactions. This information can be used by the scientific community to engineer the desired inter-helical interactions at appropriate locations in TM helices.
• The data showing conservation of a kink in proteins from the Sodium/Calcium exchanger family highlight its crucial functional role in this family. This data can be used for homology modeling of proteins within this family by computational biologists.

## 1 Data

The data used in this analysis has been generated after a detailed structural examination of membrane proteins. This structural data provides solid evidence for the utility and various roles of perturbed helices in membrane proteins. See Fig. 1, Fig. 2, Fig. 3, Fig. 4, Fig. 5, Fig. 6, Fig. 7, Fig. 8, Fig. 9, Fig. 10, Fig. 11, Fig. 12, Fig. 13, Fig. 14, Fig. 15, Fig. 16, Fig. 17 and Table 1, Table 2, Table 3, Table 4, Table 5.

## 2 Experimental design, materials and methods

Structural analysis of membrane protein structures was performed after they were downloaded from the Orientation of Proteins in Membrane (OPM) database [9]. The identification of secondary structures was carried out using Assignment of Secondary Structures in Proteins (ASSP) [10] and non-bonded interactions were identified using MolBridge [11]. Next, we identified geometries of helical fragments using Helanal-Plus [2] and computed the backbone torsion angles (φ–ψ). Multiple sequence alignment of protein sequences was carried out using ClustalΩ [12].

We prepared datasets of proteins belonging to Sodium Calcium family of transporters as mentioned in [1] to examine conservation of kinks in functionally important helices. A dataset of proteins belonging to Heme Copper Oxidase (HCO) superfamily was created to gain insights about the presence of the π-helix in each protein (Table 3). To understand the variation if any in the π-helix within different types of HCOs, we analyzed two crystal structures from the A-type, one from B-type and three crystal structures from the C-type HCOs along with proteins representing each Nitric Oxide Reductase (Table 3). The presence of the unusually long π-helix in Cytochrome-c-oxidase (PDB ID: 1v55) defined by ASSP was reconfirmed by its identification using DSSP – a program based on hydrogen bond energetics for secondary structure identification (http://www.cmbi.ru.nl/dssp.html) (Table 4).

Pair-wise crossing angles between TM helices were determined by calculating the cross products of direction cosines (l, m, n) as computed by Helanal-Plus.

## Figures and Tables

**Fig. 1:**
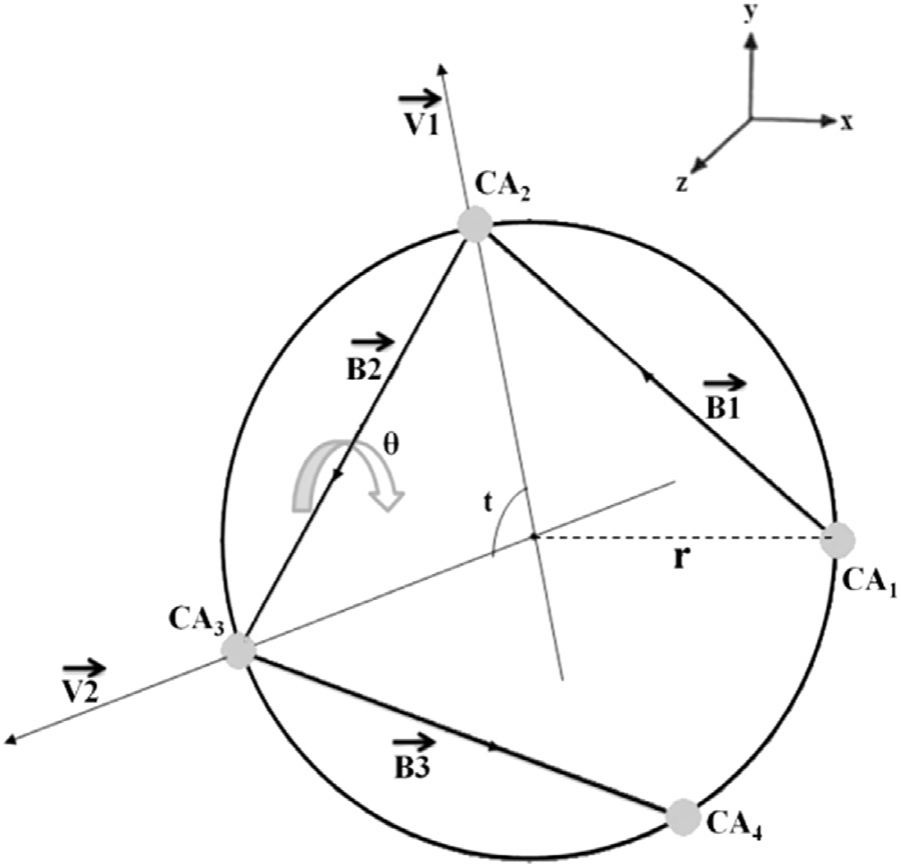
Schematic representation depicting the method used for calculation of local helix parameters. The points CA_1_, CA_2_, CA_3_, CA_4_ represent the four consecutive Cα atoms of a helix projected down the helix axis. B1, B2 and B3 are vectors joining the points CA_1_CA_2_, CA_2_CA_3_, CA_3_CA_4_ respectively. V1 and V2 are angle bisectors of the angles CA_1_CA_2_CA_3_ and CA_2_CA_3_CA_4_, respectively. The dot product of the two vectors V1 and V2 gives the twist value. The direction cosines U (l,m,n) of the helix axis are obtained from the cross products of vectors V1 and V2. The rise per residue is obtained by computing the dot product between the vector B2 and U. (Figure taken with permission from [2]).

**Fig. 2:**
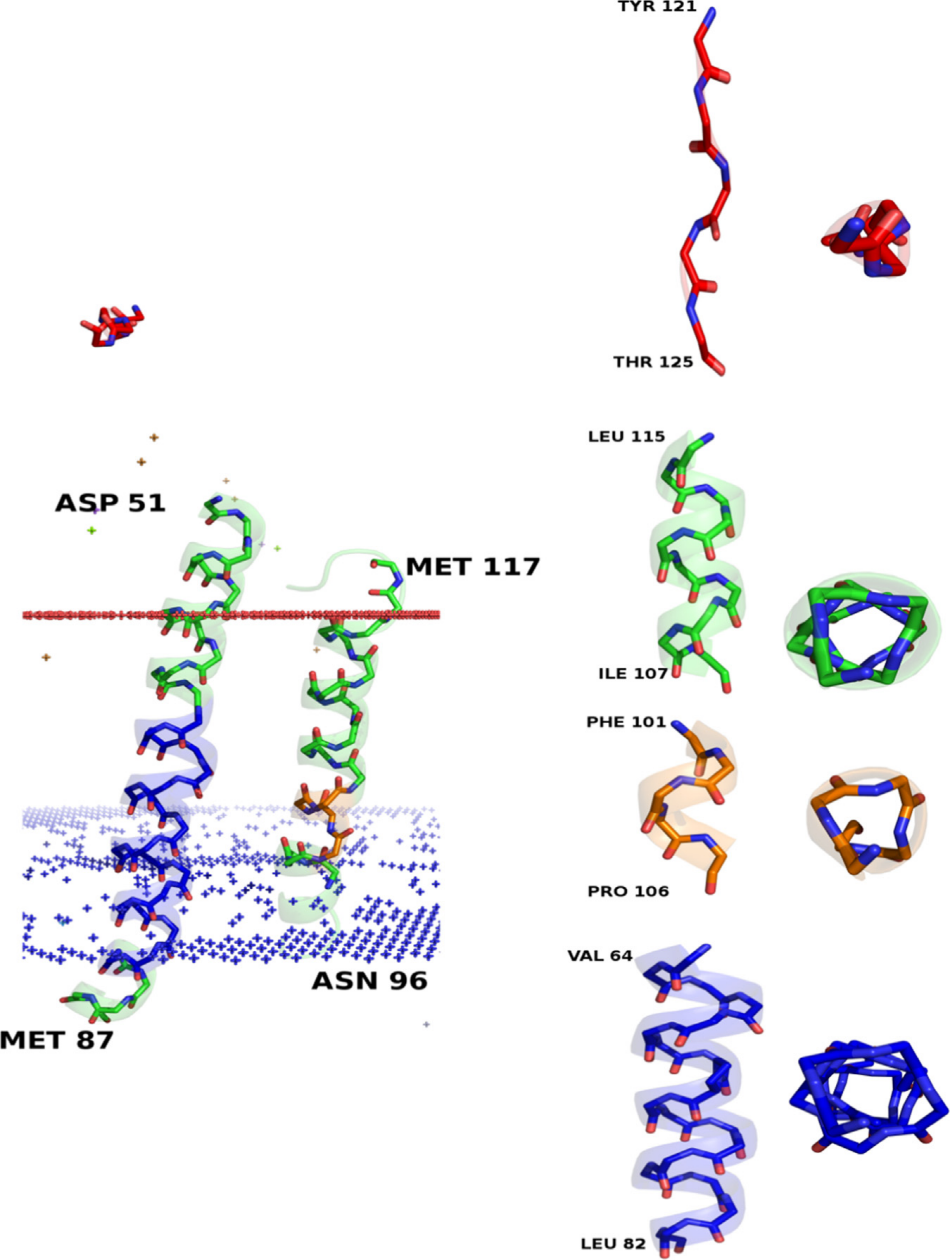
Representative examples of different types of helices identified by ASSP. α, 3_10_, π and Poly Proline II helices have been depicted in the Cytochrome-c-oxidase (PDB ID: 1v55). Enlarged front and top-down views of each helix type have also been shown.

**Fig. 3:**
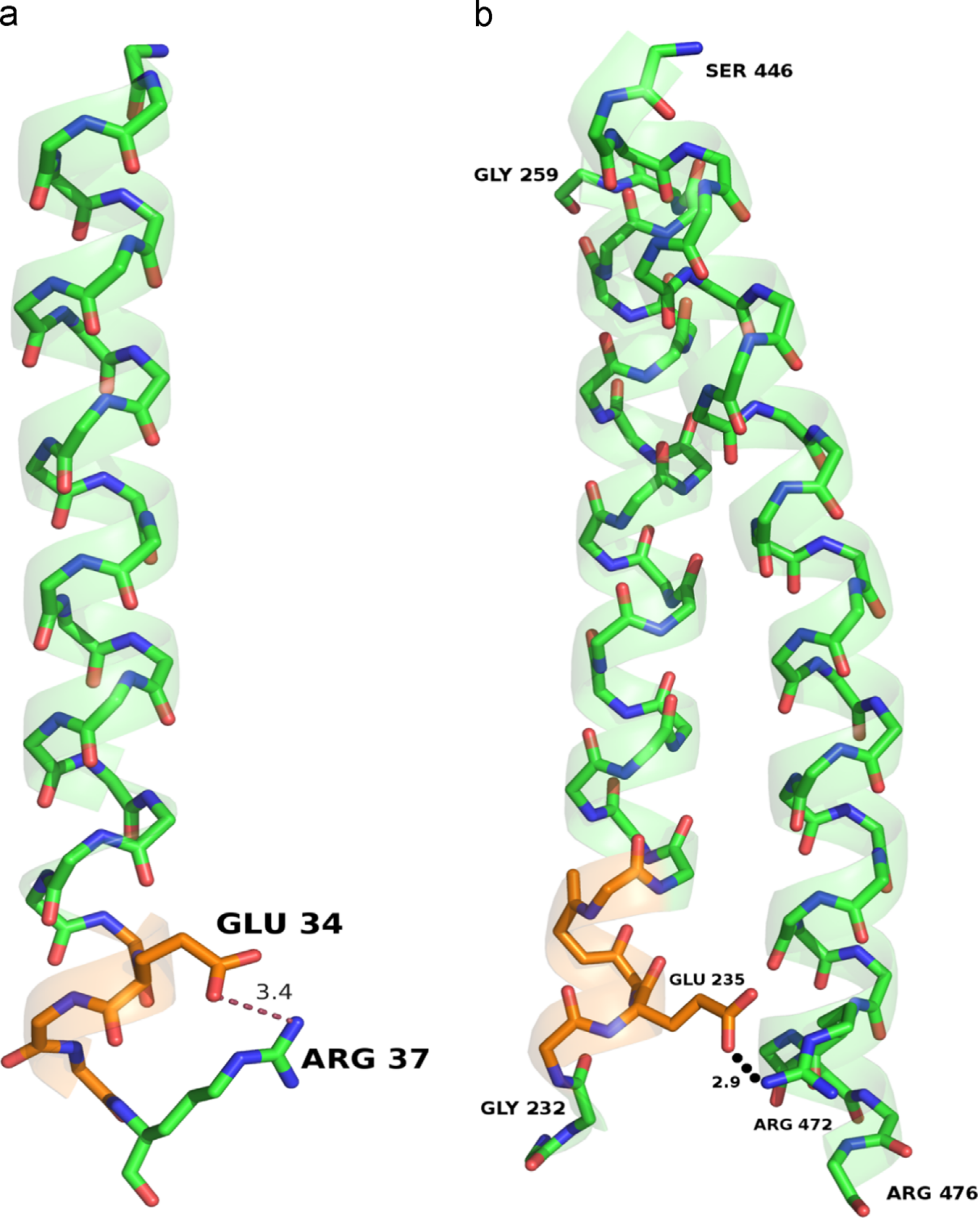
Intra and Inter-helical salt bridges stabilizing 3_10_-helices in membrane proteins. a) The side-chain of 34Glu in the 3_10_ helix (34E-36A) of TM2 in the Photosynthetic Reaction Center (PDB ID: 1rzh) forms an intra-helical salt bridge with the side-chain of 37Arg. b) The 3_10_ helix (P234-G236) of TM13 in the Photosystem II (PDB ID: 3arc) contains Glutamic acid at position 235 which forms an inter-helical salt-bridge with Arg472 from a neighboring TM helix. The depicted 3_10_ helices lie at the interfacial region and hence, membrane boundaries have not been shown for clarity.

**Fig. 4:**
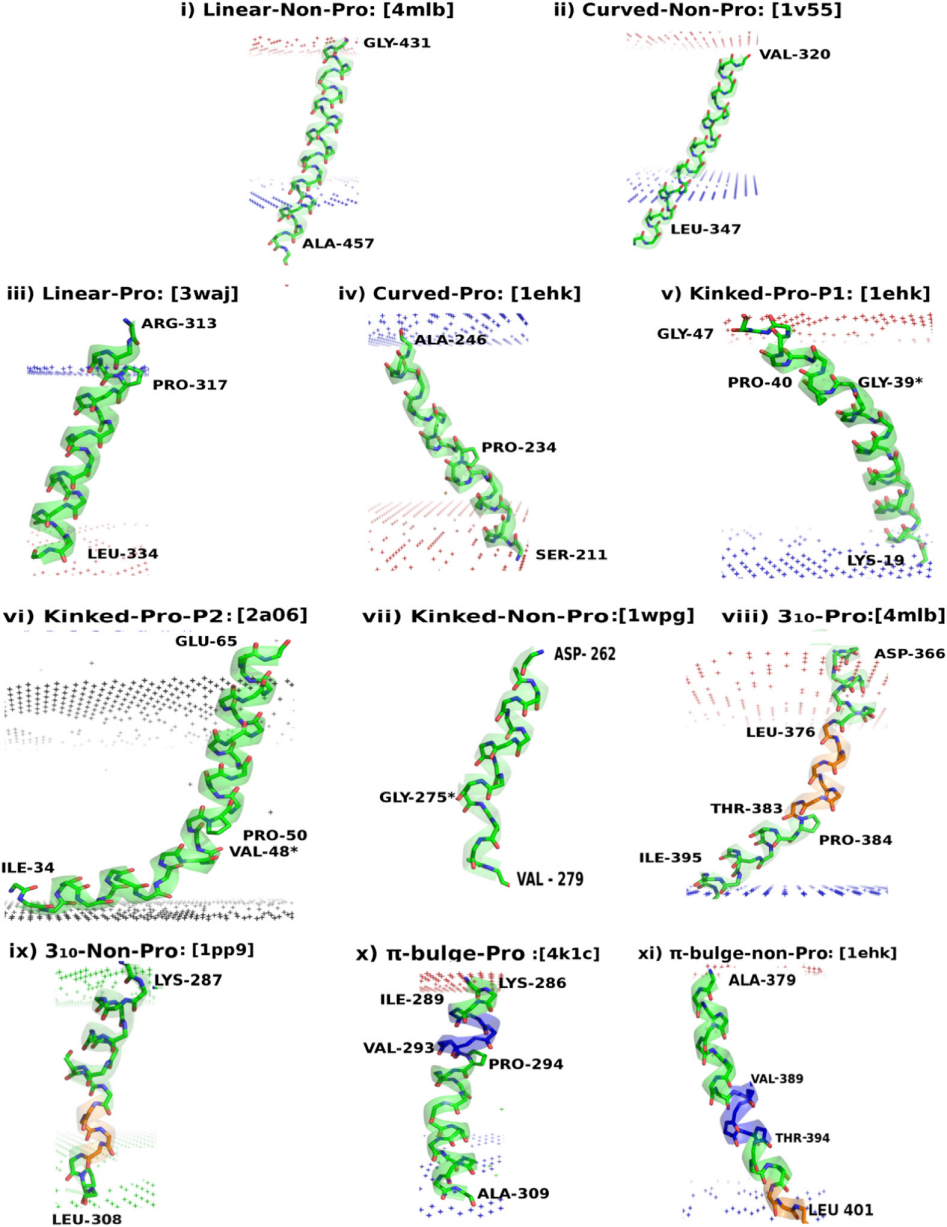
Cartoon representations of Linear and Curved helices without Proline used as reference helices (Panels i and ii) and each of the 9 types of helix perturbations (Panels iii to xi) observed in TM helices of membrane proteins. PDB identifiers are given within square braces in each panel. α, 3_10_ and π helices have been depicted in distinct colors. The ‘*’ in panels v, vi and vii denotes the residue position corresponding to maximum local bending angle.

**Fig. 5:**
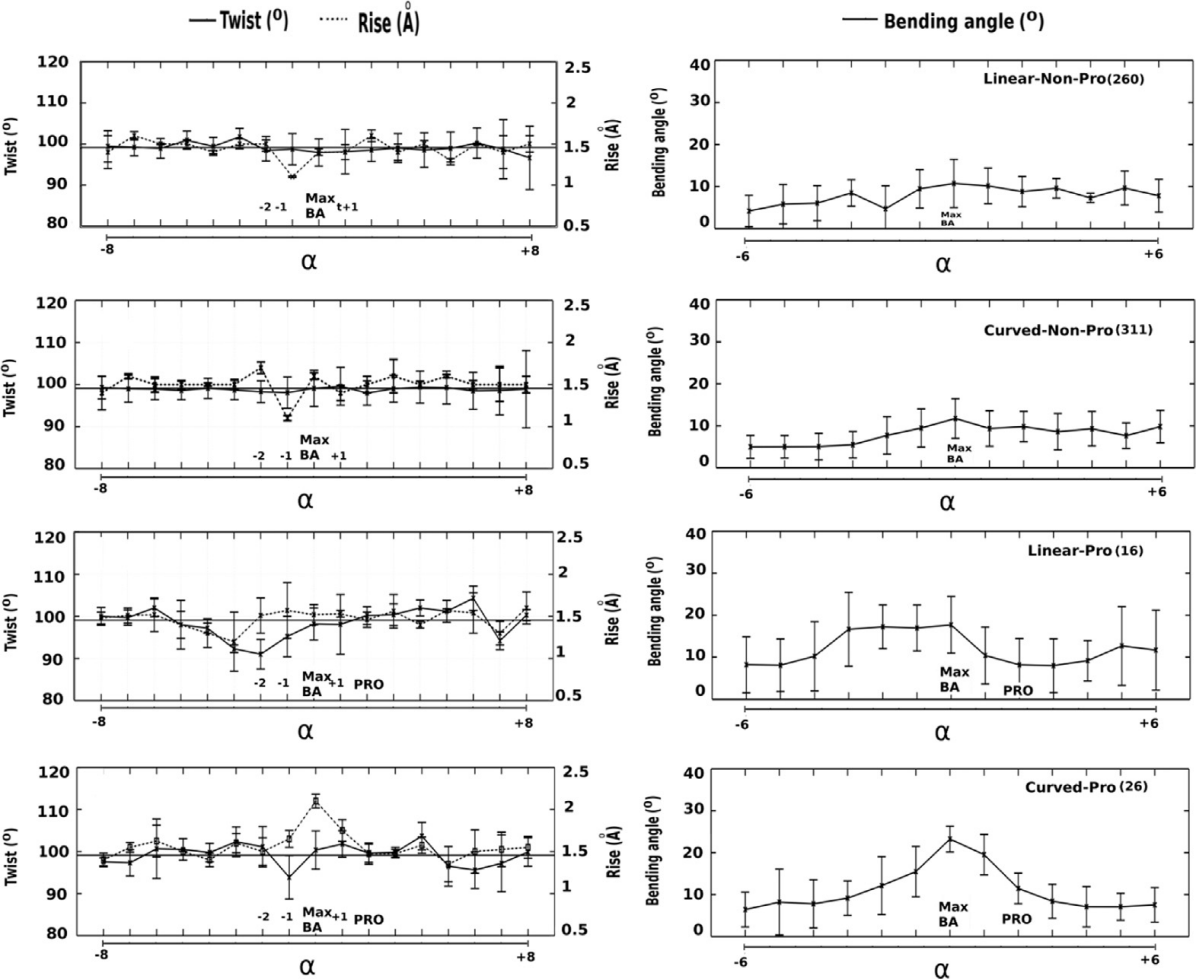
Variations in helical parameters (twist, rise per residue, local bending angles) for Linear and Curved helices with and without Proline defined by Helanal-Plus.

**Fig. 6:**
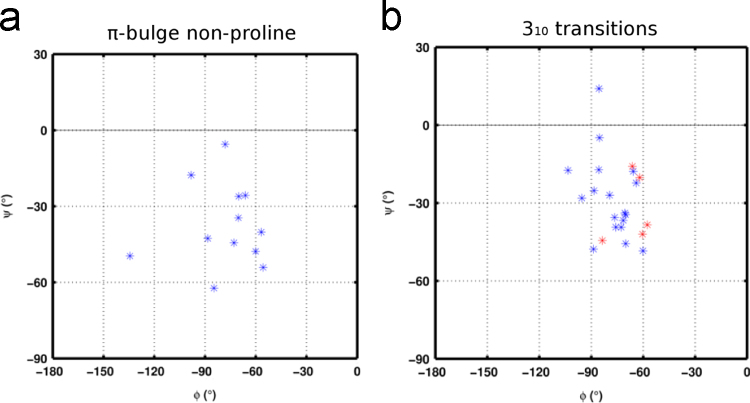
Backbone torsion angles (φ–ψ) of amino acids that have an unpaired backbone carbonyl group at −4 position relative to the residue with Maximum local bending angle (MaxBA) (see Fig. 5) of the helix perturbation. In b), the torsion angles of amino acids at −4 position in proline mediated 3_10_ transitions have been indicated in red asterisks (*).

**Fig. 7:**
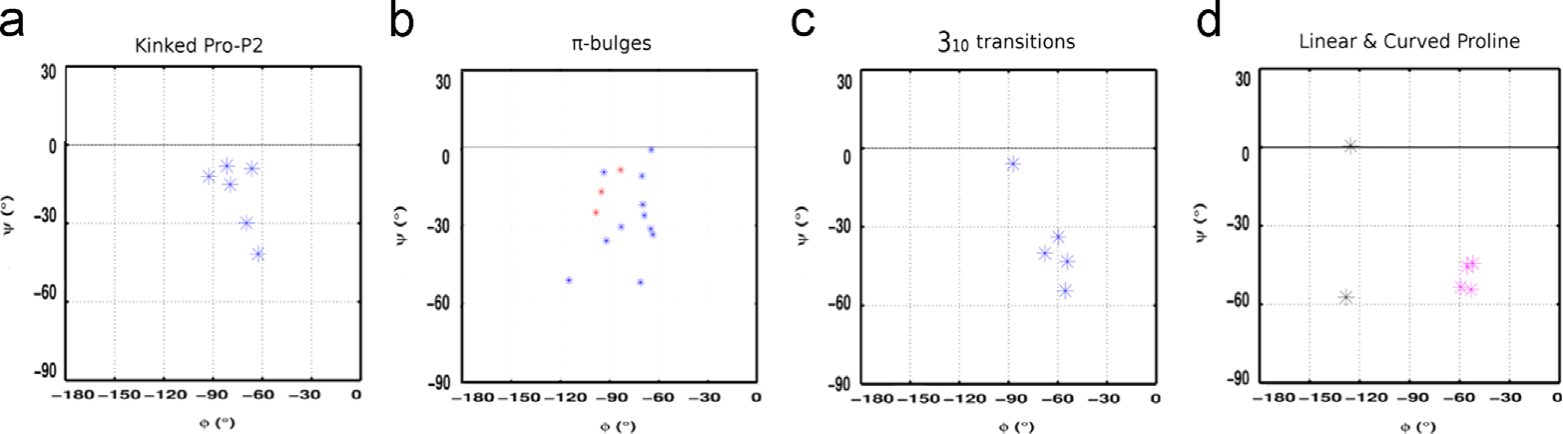
Backbone torsion angles (φ–ψ) of amino acids that have an unpaired backbone carbonyl group at -3 position relative to the residue with Maximum local bending angle (MaxBA) (see Fig. 5) of the helix perturbation. The torsion angles of amino acids at -3 position in proline mediated π-bulges (b) and 3_10_-helices (c) have been indicated in red asterisks (*) whereas those for linear and curved helices have been shown in black and magenta. (For interpretation of the references to color in this figure legend, the reader is referred to the web version of this article).

**Fig. 8:**
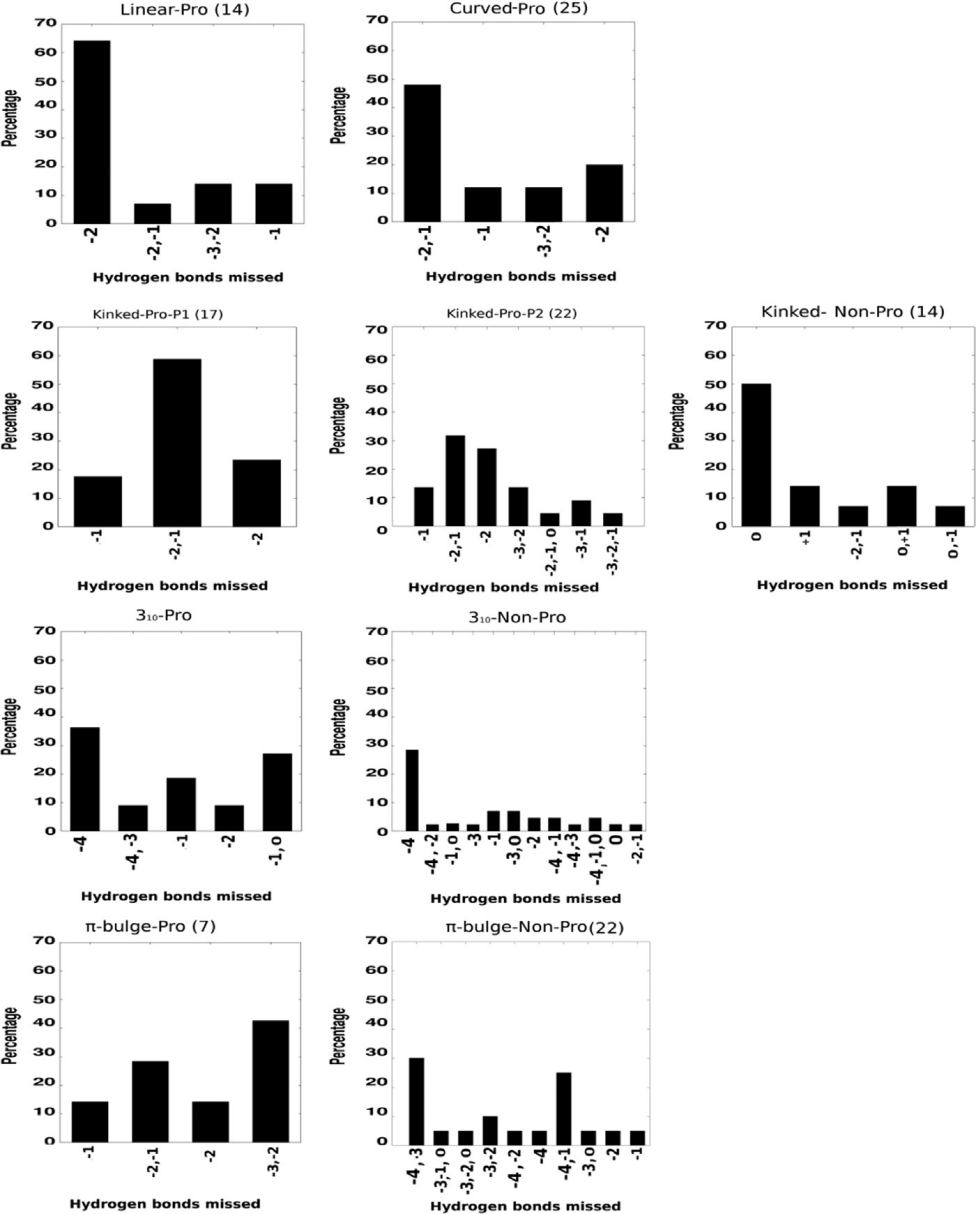
Patterns of main chain backbone carbonyl groups that are missed due to perturbations in helical regions (proline and non-proline mediated). The positions of unpaired carbonyl groups are w.r.t Proline. In the case of non-proline mediated perturbations, the carbonyl group position w.r.t the +2 position of the perturbation (see Fig. 4, Fig. 5). The numbers within parenthesis represent the cases of missed hydrogen bond for each perturbation.

**Fig. 9:**
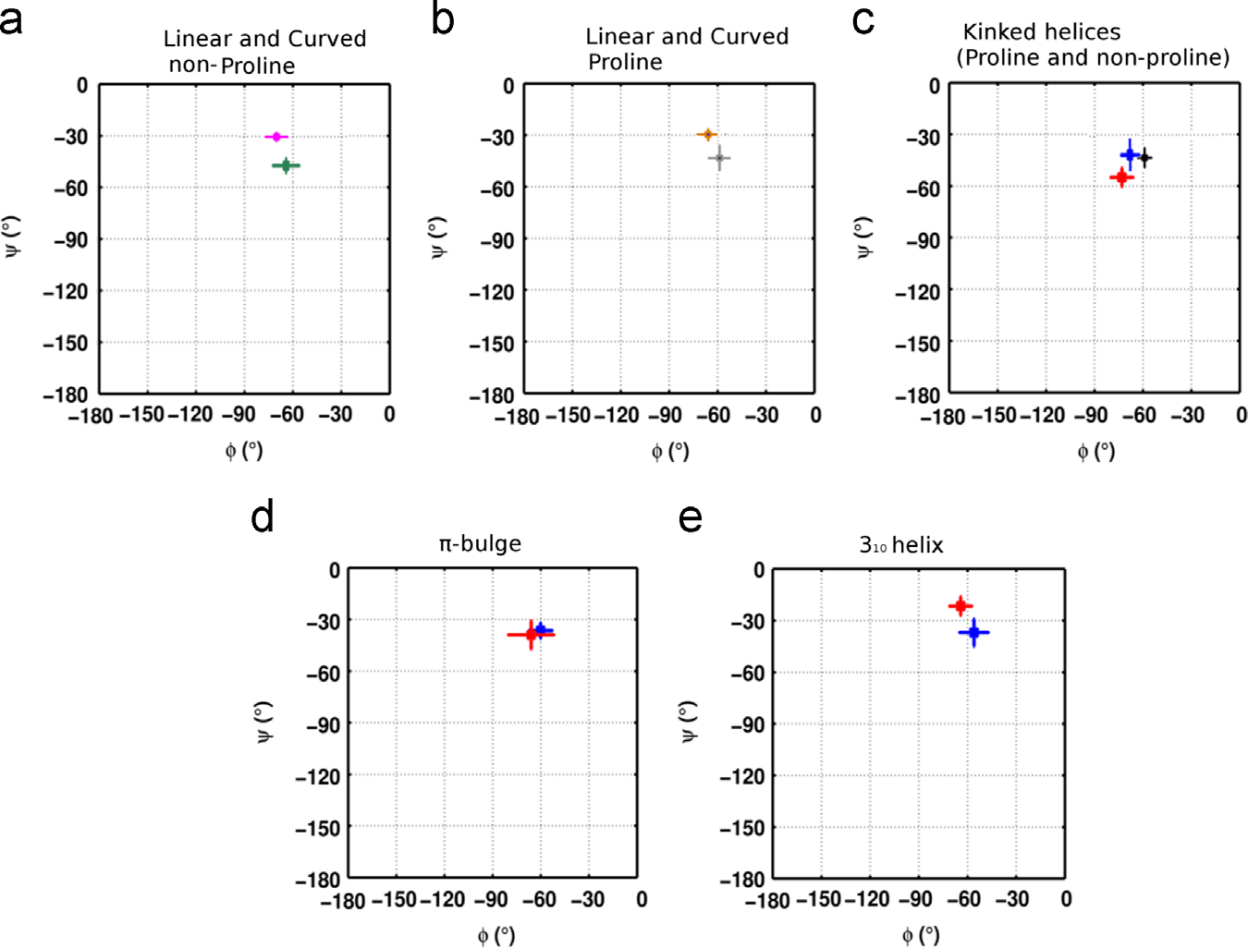
Backbone torsion angle (φ–ψ) distribution for perturbation inducing Proline or equivalent non-proline amino acid in various helix perturbations. Colour coding scheme used for representing torsion angle distributions has been adapted from Fig. 5.

**Fig. 10:**
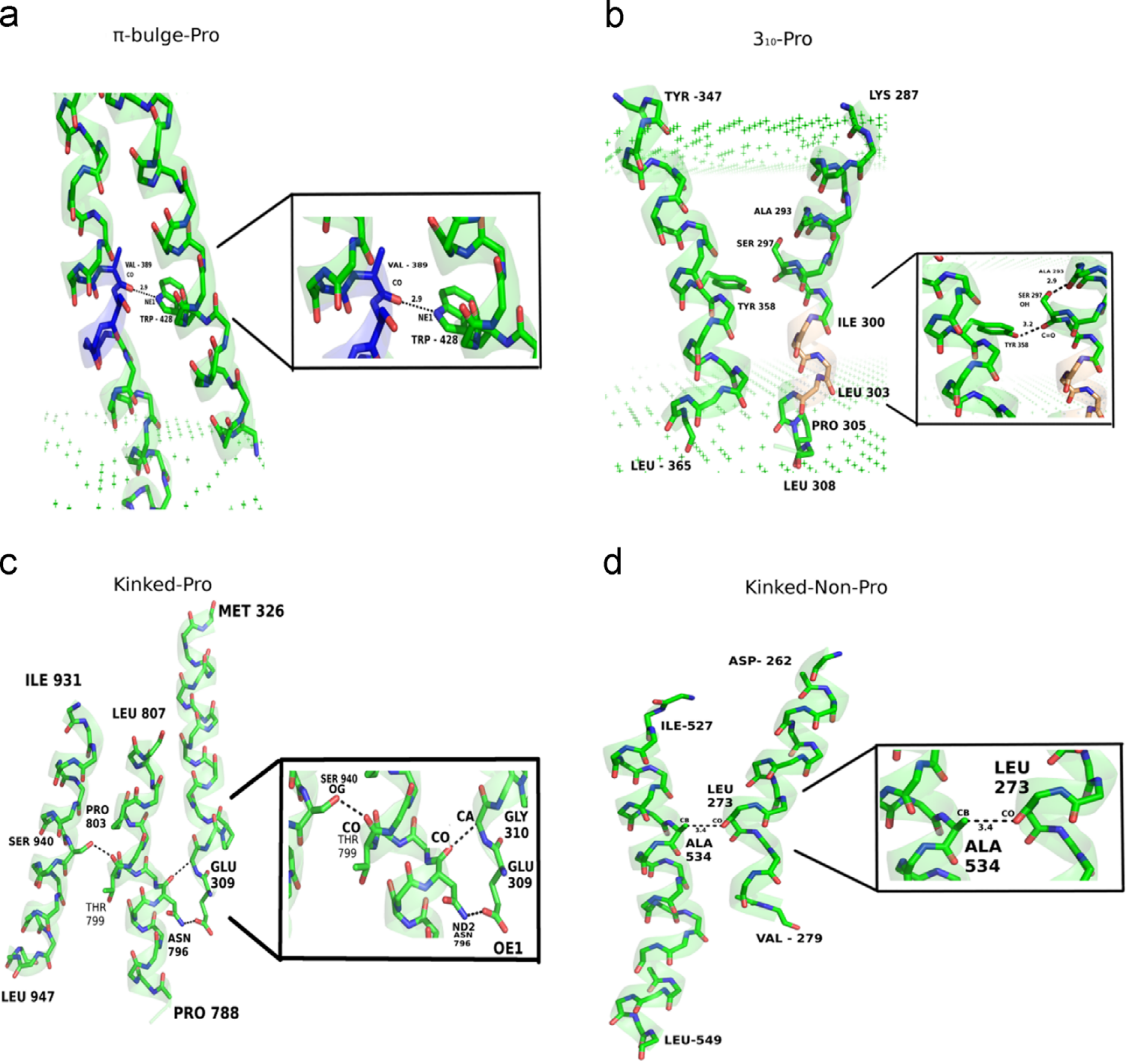
Inter and Intra-helical hydrogen bonds formed due to helix perturbations. Panels a–d illustrate examples of helix–helix interactions observed in a) Proline mediated π-bulges (Bacterial Cytochrome-c-Oxidase [1ehk]), b) Proline mediated 3_10_ helices (Bovine Cytochrome bc1 [1pp9]), c) In the Sarcoplasmic reticulum calcium ATPase (PDB ID-1wpg:A), Pro803 kinks the helical segment (788P-807L), the resulting disrupted hydrogen bonds form a network of inter-helical interactions between neighboring TM helices to stabilize the kinked helix, d) C–H...O mediated inter-helical interaction that forms TM helix contacts is depicted between two Non-Proline kinked helices in Cytochrome-c-Oxidase (PDB ID-1ehk: A). C–H...O mediated hydrogen bonds have received special attention in membrane proteins [3], [4] and several studies have elucidated their importance in other bio molecules as well [5], [6], [7], [8].

**Fig. 11:**
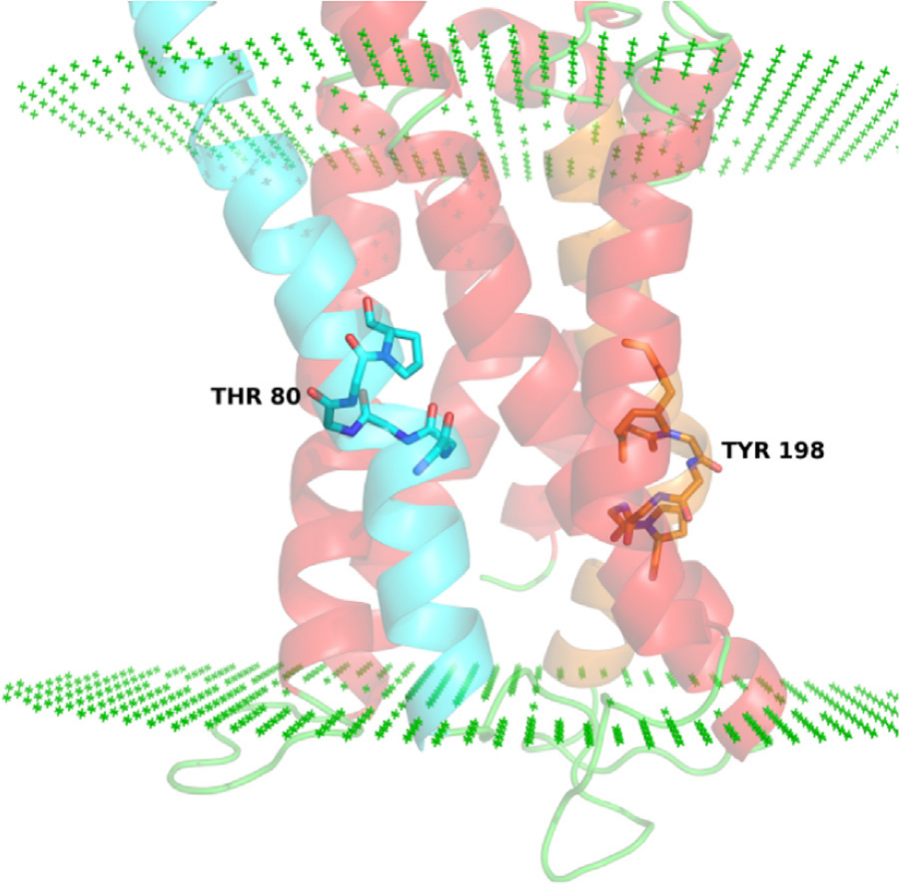
Potential role of ‘Unsatisfied’ amino acids in oligomerization of the Dopamine D2 receptor. The Proline kinked TM2 [66–91] (cyan) and TM5 [186–216] (orange) helices in the Dopamine D2 receptor have free backbone carbonyl (C=O) groups (Thr80 and Tyr198) that face the exterior of the protein. These free C=O groups have a potential role in inter-protomer hydrogen bond formation leading to higher order states/ oligomerization of the receptor. The polar side-chains of these amino can form probable inter-protomer hydrogen bonds but have not been represented for clarity. (For interpretation of the references to color in this figure legend, the reader is referred to the web version of this article).

**Fig. 12:**
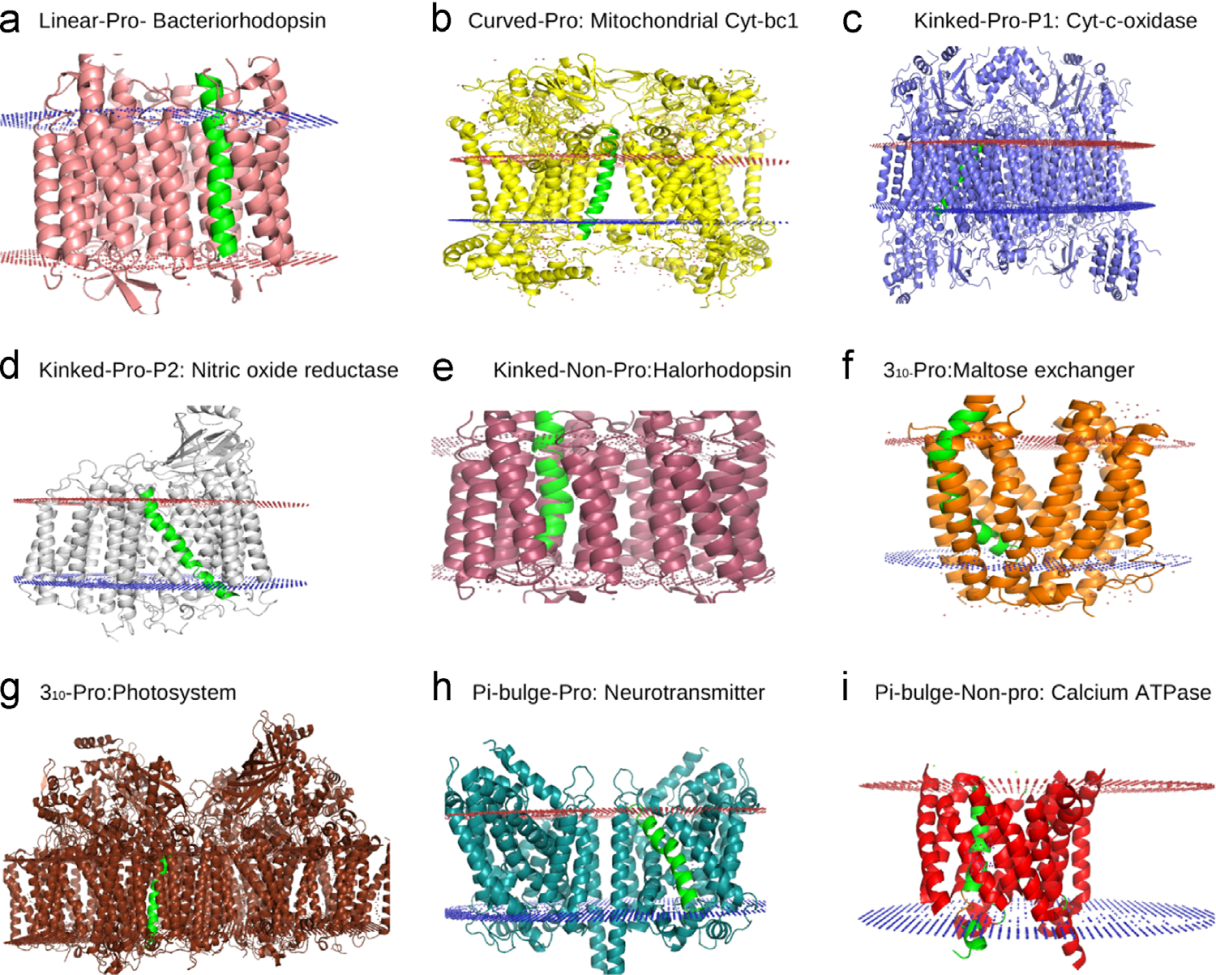
Locations of perturbed helices within the TM helix bundles. Representative examples of each TM helix perturbation (highlighted in green) observed in various membrane protein structures. Linear-Pro and Curved-Pro helices (a and b) lie near the periphery of the helix bundle hence interacting with less number of TM regions. Locations of other helix perturbations (c–i) are near the centre of the helix bundle leading to more inter-helical contacts (See Table 2). (For interpretation of the references to color in this figure legend, the reader is referred to the web version of this article) .

**Fig. 13:**
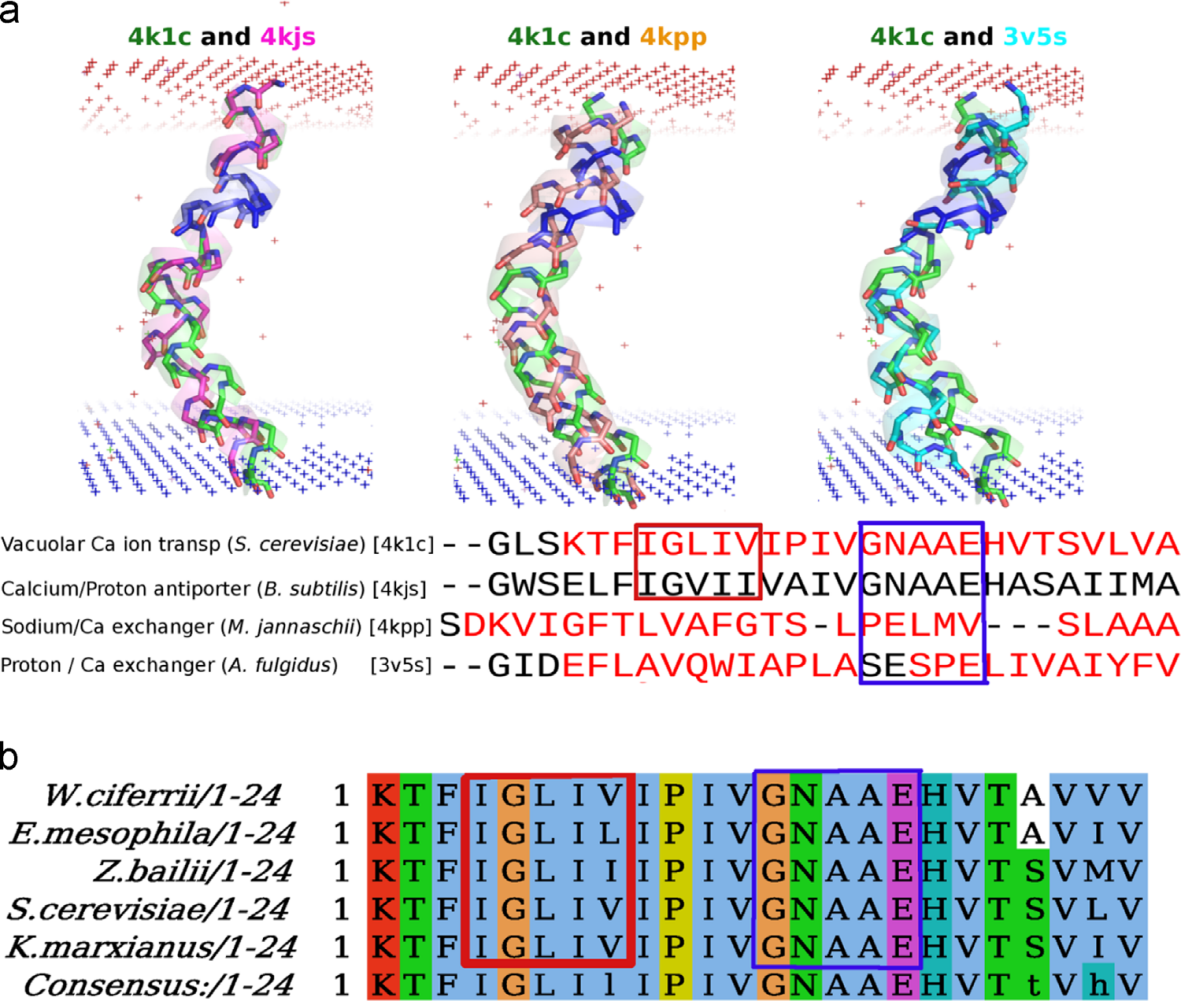
Conservation of TM helix kink in Sodium/Calcium exchanger family of proteins. a) Analysis of there related with available crystal structures (≤3.5 Å resolution) shows that the Glycine induced kink observed in the functionally important TM7 helix of the Vacuolar Calcium ion transporter [4k1c] is conserved within these distantly related protein structures of the Sodium/Calcium exchanger family despite low sequence similarity in the examined helix (blue box). The cartoon and stick representations of each TM helix has been depicted in distinct colors. The π-helix is conserved only in one family member and has been highlighted within a red box in the multiple sequence alignment. b) Sequence comparison of TM10 helix from closely related family members using BLAST shows complete conservation of the kink motif [GNAAE] (blue box) as well as the π-helix [IGLIV] (red box). (For interpretation of the references to color in this figure legend, the reader is referred to the web version of this article).

**Fig. 14:**
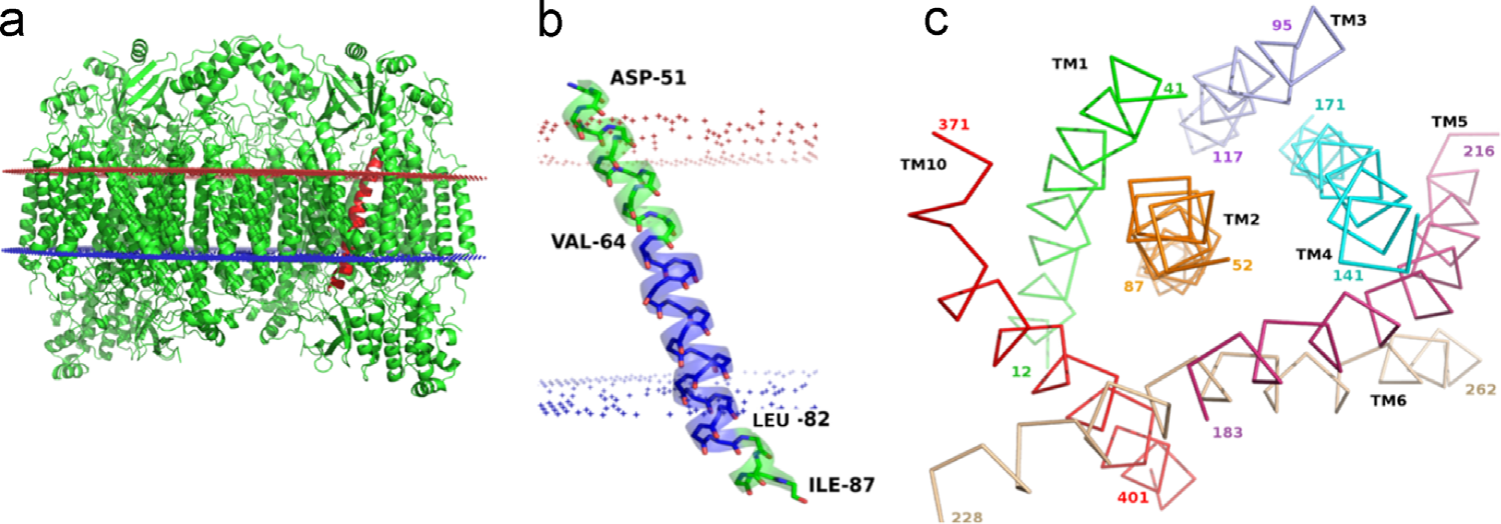
TM2 helical region (51–87) in Mitochondrial Cytochrome-c-Oxidase (1v55:A). a) Cartoon representation of Mitochondrial Cytochrome-c-Oxidase with the functionally important TM2 represented in red. b) A 19 residue long π-helix (Val64-Leu82) interspersed between two α-helical segments. c) Top-down view of ribbon representations for transmembrane helices TM1-TM6 and TM 10 indicating that TM2 (orange) is the central helix within a helical bundle. (For interpretation of the references to color in this figure legend, the reader is referred to the web version of this article).

**Fig. 15:**
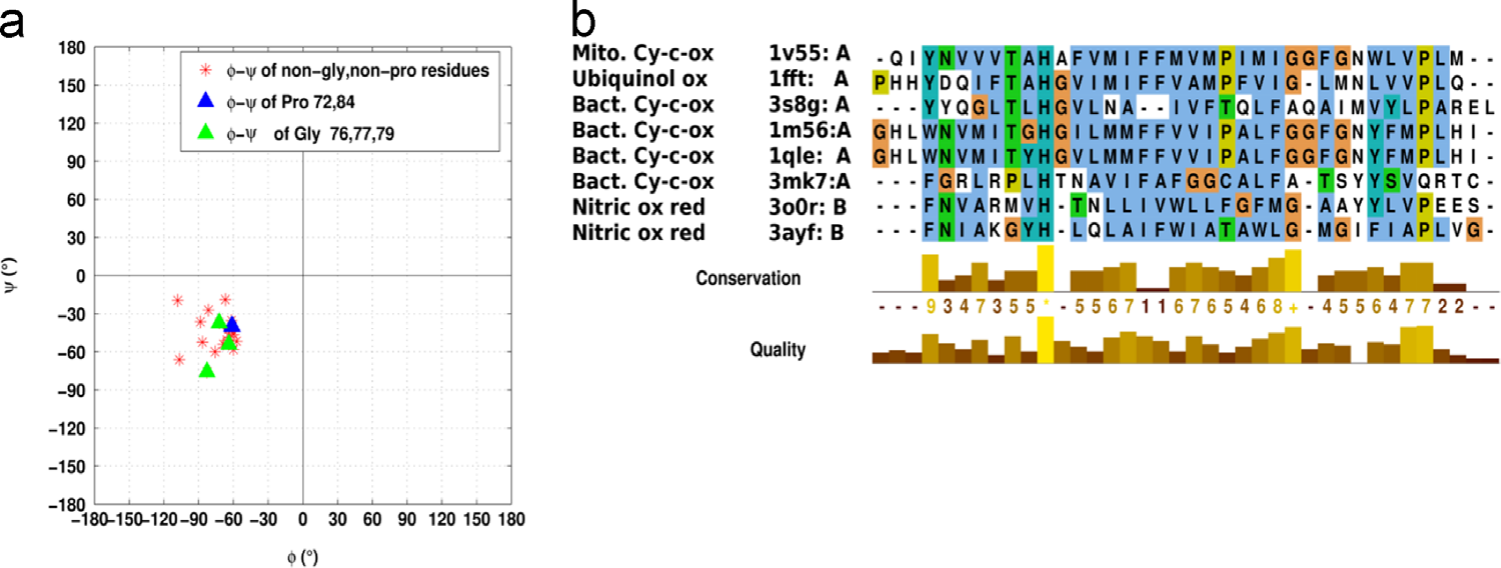
Ramachandran Map for π-helical (64–82) region in mitochondrial COX and multiple sequence alignment of the heme copper oxidase (HCO) superfamily members. a) Pro84 is not a part of the π-helix but the φ–ψ for it has been represented to show that it has similar torsion angles outside the helix perturbation as well. b) Multiple sequence alignment for the helical region analogous to TM2 of the reference protein containing the 19 residue π-helix for all HCO superfamily members (see Table 3).

**Fig. 16:**
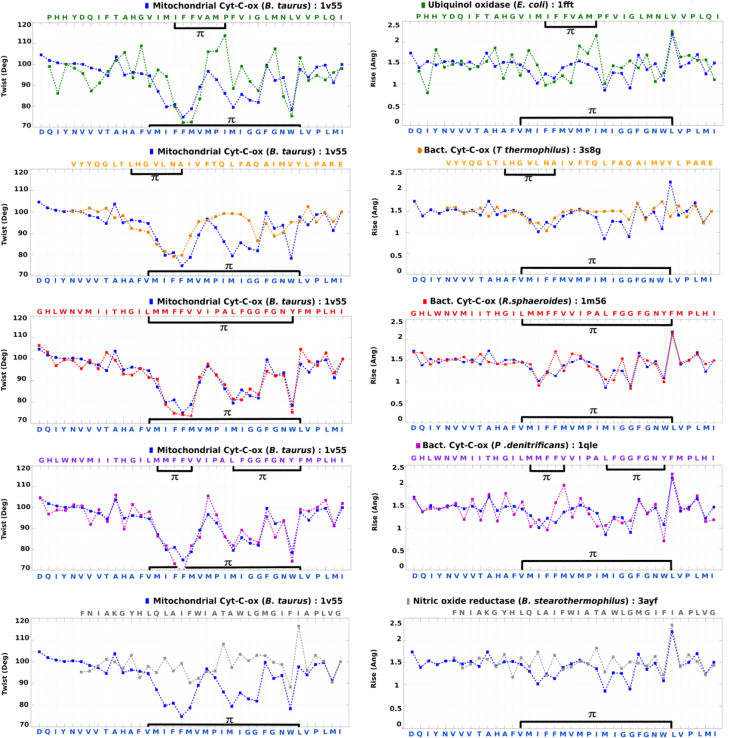
Comparison of twist and rise for TM2 region in Heme Copper Oxidase superfamily proteins. The amino acid sequence, twist and rise for the TM2 region in the reference protein has been plotted in blue, whereas the values for other superfamily members have been represented in a different colour.

**Fig. 17:**
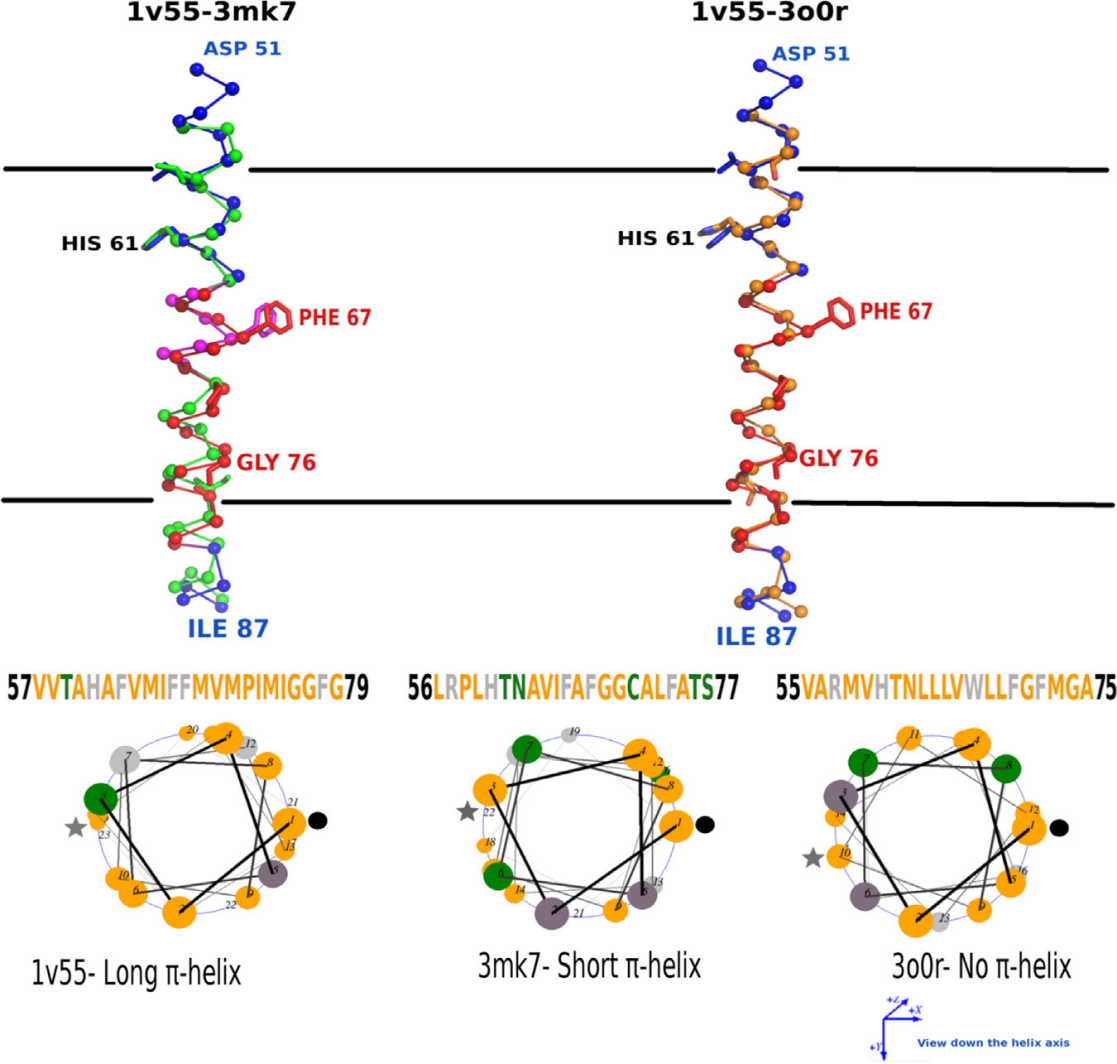
Long π-helices allow accommodation of more amino acids in the membrane. Helical regions have been represented as ribbons with C^α^ atoms highlighted as spheres. The α- and π-helical regions of the reference protein (Mitochondrial COX-1v55) have been represented in blue and red ribbons respectively. The corresponding α-helical regions of 3mk7, 3o0r and 3ayf have been shown in green, orange and grey colours. a) The bacterial COX has a small interspersed π-helix that accommodates a Phenylalanine within the helical region as observed in the reference protein. b and c) The long π-helix accommodates two extra residues (Phe67 and Gly76) in the helical region as compared to α-helices observed in NORs. The entry and exit points of the helix in the membrane have been represented as a ‘•’ and ‘*’ respectively.

**Table 1:** Occurrence of helix perturbations in various membrane protein types. Numbers within square brackets indicate the examples of different membrane protein types present in the dataset and the total number of helices within them (*italicized*). Numbers in round brackets (in bottom row) indicate the helices with perturbations occurring in a membrane protein type. ‘Other’ type of membrane proteins include all categories having individual occurrences <5.

	Transporters [*16,298*]	Channels [*7,42*]	Reductases [*5,152*]	ATPases [*5,104*]	Cyto-c-oxidases [*10,146*]	GPCRs [*9,63*]	Major. intrinsic proteins [*7,96*]	Photo systems [*6,105*]	Rhodopsins [*5,37*]	Proteases [*5,18*]	Other [*15,83*]	Total
**Linear Pro**	5	2	1	1	3	0	0	2	1	0	1	16
**Curved Pro**	6	0	8	1	3	1	1	2	3	1	0	26
**Kinked-Pro-P1**	3	1	3	2	4	1	0	1	0	1	2	18
**Kinked-Pro-P2**	6	5	0	1	4	1	0	2	1	1	1	22
**Kinked-Non-Pro**	5	3	2	2	5	1	1	2	3	0	1	26
**3**_**10**_**-Pro**	5	2	0	1	6	0	0	3	1	0	1	20
**3**_**10**_**-Non-Pro**	12	6	6	5	10	1	1	5	2	0	1	49
**π-bulge-Pro**	1	1	1	2	9	1	0	6	1	0	1	23
**π-bulge-Non-Pro**	4	1	1	3	5	2	1	4	0	1	1	23
**Total**	47 (15.7)	21 (50)	22 (14.4)	18 (18)	49 (33.5)	9 (14.2)	4 (4)	27 (25.7)	12 (32.4)	4 (22)	10 (12)	223

**Table 2:** Main chain backbone C=O atoms which lack the helical N–H...O hydrogen bond and contribute to helical interactions in each type of perturbation. Intra-helical interactions include the stabilization of the free backbone C=O atom by C^δ^ or C^γ^ atom of Proline and other intra-helical side chain to main chain (SM) hydrogen bonds. Inter-helical interactions include SM hydrogen bonds from amino acids belonging to the neighbouring helices and C^α^–H...O and C^β^–H...O hydrogen bonds. Numbers within parenthesis indicate percentage values.

**Type of Perturbation**	**No. of C=O that miss a backbone hydrogen bond**	**No. of C=O stabilized (Intra and Inter-helical hydrogen bonds)**
Linear-Pro	16	14 (87)
Curved-Pro	29	21 (72.4)
Kinked-Pro-P1	10	8 (80)
Kinked-Pro-P2	43	31 (72)
Kinked-Non-Pro	17	14 (82)
3_10-_ Pro	16	12 (75)
3_10-_ Non- Pro	65	51 (78.4)
π-bulge-Pro	28	23 (82)
π-bulge- Non- Pro	14	12 (85)
Total	218	186 (85.3)

**Table 3:** Proteins from the Heme-Copper Oxidase (HCO) superfamily considered for the analysis of the π-helical region. A total of 8 proteins (at least one member of a particular HCO subtype) have been selected for analysis. The ‘Mitochondrial COX (1v55:A)’ belongs to the initial dataset of 90 proteins used for analysis and contains the interspersed 19 residue long π-helix. The ‘Helical region’ (fifth column) represents the entire TM segment considered for analysis. The ‘Helix assignment’ (sixth column) includes the helix boundaries for α and π-helices defined by ASSP (see methods).

**Protein**	**HCO/NOR type**	**Organism**	**Resolution**	**Helical region**	**Helix assignment**
Mitochondrial cytochrome-c-oxidase (1v55:A)	HCO–A	*B. taurus*	1.9	51–87 (37)	51–63=α, 64–82=π, 83–87=α
Ubiquinol oxidase (1fft:A)	HCO–A	*E. coli*	3.5	96–131 (36)	97–110= α, 111–117= π, 118–131= α
Bacterial cytochrome-c-oxidase (3s8g:A)	HCO–B	*T.thermophilus*	1.8	65–97 (33)	65–71= α, 72–80=π, 81=97= α
Bacterial cytochrome-c-oxidase (1m56:A)	HCO–C	*R. sphaeroides*	2.3	92–128 (37)	92–104= α, 105–122=π, 123–128= α
Bacterial cytochrome-c-oxidase (1qle:A)	HCO–C	*P. denitrificans*	3.0	84–120 (37)	84–97= α, 98–102=π, 103–106= α, 107–115= π, 116–120= α
Bacterial cytochrome-c-oxidase (3mk7:A)	HCO–C	*P. stutzeri*	3.2	53–85 (33)	53–62= α, 63–69= π, 70–84= α
Nitric oxide reductase (3o0r:B)	cNOR	*P. aeruginosa*	2.7	53–84 (32)	53–84= α
Nitric oxide reductase (3ayf:A)	qNOR	*B. stearothermo philus*	2.5	348–379 (32)	348–379= α

**Table 4:** Tabulated output files of ASSP and DSSP defining the long π-helical region in mitochondrial COX. ASSP defines a π-helix from (64 V-82 L) based on twist, rise per residue and helical radius whereas DSSP defines a π-helix from (64 V-79 G) denoted by the symbol ‘I’ based on backbone hydrogen bond energetics.

**ASSP OUTPUT**									
**HELIX STEP**					**TWIST**	**RISE**	**VTOR**	**BEND**	**RADIUS**
**51**	**51 D**	**52 Q**	**53 I**	**54 Y A**	**101.9**	**1.4**	**48.0**	**94.0**	**2.3**
**52**	**52 Q**	**53 I**	**54 Y**	**55 N A**	**100.7**	**1.5**	**52.2**	**166.5**	**2.3**
**53**	**53 I**	**54 Y**	**55 N**	**56 V A**	**100.1**	**1.4**	**48.6**	**9.5**	**2.3**
**54**	**54 Y**	**55 N**	**56 V**	**57 V A**	**100.4**	**1.5**	**51.6**	**3.6**	**2.3**
**55**	**55 N**	**56 V**	**57 V**	**58 V A**	**100.0**	**1.5**	**51.3**	**3.4**	**2.3**
**56**	**56 V**	**57 V**	**58 V**	**59 T A**	**98.3**	**1.5**	**47.5**	**3.9**	**2.3**
**57**	**57 V**	**58 V**	**59 T**	**60 A A**	**97.3**	**1.5**	**48.6**	**2.5**	**2.3**
**58**	**58 V**	**59 T**	**60 A**	**61 H A**	**94.6**	**1.4**	**43.8**	**2.2**	**2.4**
**59**	**59 T**	**60 A**	**61 H**	**62 A A**	**103.7**	**1.7**	**60.1**	**7.8**	**2.2**
**60**	**60 A**	**61 H**	**62 A**	**63 F A**	**94.9**	**1.4**	**44.2**	**11.8**	**2.4**
**61**	**61 H**	**62 A**	**63 F**	**64 V A**	**96.2**	**1.5**	**47.6**	**12.0**	**2.3**
**62**	**62 A**	**63 F**	**64 V**	**65 M A**	**95.6**	**1.5**	**47.7**	**8.8**	**2.4**
**63**	**63 F**	**64 V**	**65 M**	**66 I A**	**94.6**	**1.5**	**45.0**	**3.8**	**2.4**
**64**	**64 V**	**65 M**	**66 I**	**67 F A**	**87.1**	**1.3**	**36.0**	**4.1**	**2.6**
**65**	**65 M**	**66 I**	**67 F**	**68 F A**	**79.7**	**1.0**	**25.1**	**5.8**	**2.8**
**66**	**66 I**	**67 F**	**68 F**	**69 M A**	**80.9**	**1.2**	**31.3**	**9.9**	**2.8**
**67**	**67 F**	**68 F**	**69 M**	**70 V A**	**74.8**	**1.1**	**25.9**	**4.9**	**3.0**
**68**	**68 F**	**69 M**	**70 V**	**71 M A**	**78.8**	**1.4**	**33.4**	**0.2**	**2.8**
**69**	**69 M**	**70 V**	**71 M**	**72 P A**	**89.2**	**1.5**	**41.6**	**6.1**	**2.5**
**70**	**70 V**	**71 M**	**72 P**	**73 I A**	**96.6**	**1.5**	**49.0**	**4.1**	**2.3**
**71**	**71 M**	**72 P**	**73 I**	**74 M A**	**92.7**	**1.5**	**44.1**	**4.4**	**2.4**
**72**	**72 P**	**73 I**	**74 M**	**75 I A**	**86.1**	**1.4**	**37.0**	**4.9**	**2.6**
**73**	**73 I**	**74 M**	**75 I**	**76 G A**	**79.5**	**0.8**	**21.1**	**9.2**	**2.9**
**74**	**74 M**	**75 I**	**76 G**	**77 G A**	**85.6**	**1.3**	**34.5**	**14.4**	**2.6**
**75**	**75 I**	**76 G**	**77 G**	**78 F A**	**82.8**	**1.2**	**32.5**	**12.2**	**2.7**
**76**	**76 G**	**77 G**	**78 F**	**79 G A**	**81.8**	**0.9**	**23.5**	**15.5**	**2.8**
**77**	**77 G**	**78 F**	**79 G**	**80 N A**	**99.7**	**1.7**	**55.5**	**19.8**	**2.2**
**78**	**78 F**	**79 G**	**80 N**	**81 W A**	**92.3**	**1.3**	**40.4**	**19.5**	**2.5**
**79**	**79 G**	**80 N**	**81 W**	**82 L A**	**93.7**	**1.5**	**45.2**	**19.3**	**2.4**
**80**	**80 N**	**81 W**	**82 L**	**83 V A**	**78.3**	**1.1**	**26.5**	**4.3**	**2.9**
**81**	**81 W**	**82 L**	**83 V**	**84 P A**	**97.7**	**2.2**	**67.3**	**20.8**	**2.0**
**82**	**82 L**	**83 V**	**84 P**	**85 L A**	**93.9**	**1.4**	**43.0**	**27.2**	**2.4**
**83**	**83 V**	**84 P**	**85 L**	**86 M A**	**98.7**	**1.5**	**48.8**	**31.9**	**2.3**
**84**	**84 P**	**85 L**	**86 M**	**87 I A**	**99.6**	**1.7**	**55.7**	**25.6**	**2.2**
**85**	**85 L**	**86 M**	**87 I**	**88 G A**	**91.3**	**1.2**	**36.6**	**15.0**	**2.5**
**86**	**86 M**	**87 I**	**88 G**	**89 A A**	**227.9**	**2.6**	**247.0**	**94.2**	**1.5**

**DSSP OUTPUT**								
**RESIDUE**	**AA STRUCTURE**	**BP1**	**BP2**	**ACC**	**N-H—>O**	**O-->H-N**	**N-H-->O**	**O-->H-N**

**51**	**51 A D H**	**3> S+**	**0 0**	**74**	**-2,-0.3**	**4,-2.3**	**1,-0.2**	**5,-0.1**
**52**	**52 A Q H**	**3> S+**	**0 0**	**58**	**2,-0.2**	**4,-1.9**	**1,-0.2**	**-1,-0.2**
**53**	**53 A I H**	**<> S+**	**0 0**	**76**	**-3,-0.5**	**4,-2.3**	**2,-0.2**	**-2,-0.2**
**54**	**54 A Y H**	**X S+**	**0 0**	**2**	**-4,-1.8**	**4,-2.3**	**1,-0.2**	**-2,-0.2**
**55**	**55 A N H**	**X S+**	**0 0**	**49**	**-4,-2.3**	**4,-2.2**	**1,-0.2**	**70,-0.4**
**56**	**56 A V H**	**X S+**	**0 0**	**18**	**-4,-1.9**	**4,-2.7**	**67,-0.2**	**-1,-0.2**
**57**	**57 A V H**	**X S+**	**0 0**	**15**	**-4,-2.3**	**4,-2.3**	**2,-0.2**	**-2,-0.2**
**58**	**58 A V H**	**X S+**	**0 0**	**27**	**-4,-2.3**	**4,-1.8**	**2,-0.2**	**-2,-0.2**
**59**	**59 A T H**	**X S+**	**0 0**	**4**	**-4,-2.2**	**4,-2.0**	**1,-0.2**	**-2,-0.2**
**60**	**60 A A H**	**X S+**	**0 0**	**13**	**-4,-2.7**	**4,-2.8**	**1,-0.2**	**5,-0.3**
**61**	**61 A H H**	**X S+**	**0 0**	**24**	**-4,-2.3**	**4,-2.4**	**-5,-0.2**	**-1,-0.2**
**62**	**62 A A H**	**X S+**	**0 0**	**17**	**-4,-1.8**	**4,-2.4**	**2,-0.2**	**5,-0.3**
**63**	**63 A F H**	**X>S+**	**0 0**	**5**	**-4,-2.0**	**4,-2.2**	**1,-0.2**	**5,-0.8**
**64**	**64 A V I**	**X>S+**	**0 0**	**0**	**-4,-2.8**	**5,-1.5**	**3,-0.2**	**4,-1.4**
**65**	**65 A M I**	**<>S+**	**0 0**	**40**	**-4,-2.4**	**5,-2.9**	**-5,-0.3**	**-2,-0.2**
**66**	**66 A I I**	**<>S+**	**0 0**	**14**	**-4,-2.4**	**5,-2.0**	**-5,-0.2**	**-2,-0.2**
**67**	**67 A F I**	**<5S+**	**0 0**	**15**	**-4,-2.2**	**-3,-0.2**	**-5,-0.3**	**-2,-0.1**
**68**	**68 A F I**	**<<S+**	**0 0**	**2**	**-4,-1.4**	**-42,-0.3**	**-5,-0.8**	**-41,-0.2**
**69**	**69 A M I**	**><S+**	**0 0**	**15**	**-5,-1.5**	**4,-2.0**	**-6,-0.2**	**5,-0.2**
**70**	**70 A V I**	**><S+**	**0 0**	**8**	**-5,-2.9**	**4,-2.6**	**1,-0.2**	**5,-0.3**
**71**	**71 A M H**	**>XS+**	**0 0**	**7**	**-5,-2.0**	**4,-1.9**	**1,-0.2**	**5,-1.3**
**72**	**72 A P I**	**4>S+**	**0 0**	**3**	**0, 0.0**	**5,-1.7**	**0, 0.0**	**-1,-0.2**
**73**	**73 A I I**	**<>S+**	**0 0**	**14**	**-4,-2.0**	**5,-2.7**	**3,-0.2**	**6,-0.3**
**74**	**74 A M I**	**<>S+**	**0 0**	**2**	**-4,-2.6**	**5,-1.0**	**-5,-0.2**	**-3,-0.2**
**75**	**75 A I I**	**<>S+**	**0 0**	**17**	**-4,-1.9**	**5,-0.7**	**-5,-0.3**	**-2,-0.1**
**76**	**76 A G I**	**<S+**	**0 0**	**3**	**-5,-1.3**	**4,-0.4**	**3,-0.2**	**-57,-0.2**
**77**	**77 A G I**	**><S+**	**0 0**	**0**	**-5,-1.7**	**4,-2.3**	**-6,-0.3**	**5,-0.2**
**78**	**78 A F I**	**><S+**	**0 0**	**0**	**-5,-2.7**	**4,-2.7**	**1,-0.2**	**5,-0.4**
**79**	**79 A G I**	**><S+**	**0 0**	**0**	**-5,-1.0**	**4,-2.3**	**-6,-0.3**	**-3,-0.2**
**80**	**80 A N H**	**4<S+**	**0 0**	**0**	**-5,-0.7**	**5,-0.2**	**-4,-0.4**	**-2,-0.2**
**81**	**81 A W H**	**X S+**	**0 0**	**8**	**-4,-2.3**	**4,-0.6**	**-5,-0.1**	**-2,-0.2**
**82**	**82 A L H**	**X S+**	**0 0**	**0**	**-4,-2.7**	**4,-2.8**	**-5,-0.2**	**-3,-0.2**
**83**	**83 A V H**	**X S+**	**0 0**	**0**	**-4,-2.3**	**4,-1.0**	**-5,-0.4**	**6,-0.2**
**84**	**84 A P H**	**>4>S+**	**0 0**	**0**	**0, 0.0**	**5,-2.6**	**0, 0.0**	**3,-0.7**
**85**	**85 A L H**	**><5S+**	**0 0**	**1**	**-4,-0.6**	**3,-0.9**	**1,-0.2**	**-2,-0.2**
**86**	**86 A M H**	**3<5S+**	**0 0**	**4**	**-4,-2.8**	**-1,-0.2**	**1,-0.2**	**406,-0.2**
**87**	**87 A I T**	**<<5S-**	**0 0**	**1**	**-4,-1.0**	**-1,-0.2**	**-3,-0.7**	**-2,-0.2**

**Table 5:** Pair wise crossing angles for helices in the vicinity of the TM2 (reference protein)/ and structurally equivalent helix in HCO superfamily proteins. Numbering scheme of the TM helices belongs to helices in the reference protein, corresponding helical regions have been considered from other members of the HCO family. Crossing angle values for helical regions that do not interact with the TM2/ structurally equivalent helix have been italicized and underscored.

**Protein**	**Helices in vicinity**					
	**TM1**	**TM3**	**TM4**	**TM5**	**TM6**	**TM10**
MitochondrialCOX (1v55:A)	155.8	170	15.1	*48.7*	45.9	37.5
Ubiquinol oxidase (1fft:A)	157	161	14.3	*41*	47.3	35
Bacterial COX (3s8g:A)	152	*173.3*	17.2	35	26.7	*39*
Bacterial COX (1m56:A)	154.6	167	16.3	30	44.2	39.4
Bacterial COX (1qle:A)	140	160	13.2	*46*	43.1	28.2
Bacterial COX (3mk7:A)	160	152	17	24	*44.6*	*46.7*
Nitric oxide reductase (3o0r:B)	*157*	170.3	13.5	32	46.2	48.3
Nitric oxide reductase (3ayf:A)	161	172	17.2	29	35	43
