# Supplementary material for: Data on diverse roles of helix perturbations in membrane proteins
Source: Data Brief. 2016 Nov 1;9:781–802. doi: 10.1016/j.dib.2016.10.023 (PMC5099277; doi:10.1016/j.dib.2016.10.023)
Supplement: Supplementary file 2 — Supplementary material [file mmc2.zip › dib/Supplementary_Table1.docx]

**Table 1: Occurrence of helix perturbations in various membrane protein types**

Numbers within square brackets indicate the examples of different membrane protein types present in the dataset and the total number of helices within them (*italicized*). Numbers in round brackets (in bottom row) indicate the helices with perturbations occurring in a membrane protein type. ‘Other’ type of membrane proteins include all categories having individual occurrences <5.

|  | Transporters [16,*298*] | Channels [7,*42*] | Reductases [5,*152*] | ATPases [5,*104*] | Cyto-c-oxidases [10,*146*] | GPCRs [9,*63*] | Major. Intrinsic Proteins [7,*96*] | Photo  systems [6,*105*] | Rhodopsins [5,*37*] | Proteases [5,*18*] | Other [15,*83*] | Total |
| --- | --- | --- | --- | --- | --- | --- | --- | --- | --- | --- | --- | --- |
| **Linear Pro** | 5 | 2 | 1 | 1 | 3 | 0 | 0 | 2 | 1 | 0 | 1 | 16 |
| **Curved Pro** | 6 | 0 | 8 | 1 | 3 | 1 | 1 | 2 | 3 | 1 | 0 | 26 |
| **Kinked-Pro-P1** | 3 | 1 | 3 | 2 | 4 | 1 | 0 | 1 | 0 | 1 | 2 | 18 |
| **Kinked-Pro-P2** | 6 | 5 | 0 | 1 | 4 | 1 | 0 | 2 | 1 | 1 | 1 | 22 |
| **Kinked-Non-Pro** | 5 | 3 | 2 | 2 | 5 | 1 | 1 | 2 | 3 | 0 | 1 | 26 |
| **3_10_-Pro** | 5 | 2 | 0 | 1 | 6 | 0 | 0 | 3 | 1 | 0 | 1 | 20 |
| **3_10_-Non-Pro** | 12 | 6 | 6 | 5 | 10 | 1 | 1 | 5 | 2 | 0 | 1 | 49 |
| **π-bulge-Pro** | 1 | 1 | 1 | 2 | 9 | 1 | 0 | 6 | 1 | 0 | 1 | 23 |
| **π-bulge-Non-Pro** | 4 | 1 | 1 | 3 | 5 | 2 | 1 | 4 | 0 | 1 | 1 | 23 |
| **Total** | 47 (15.7) | 21 (50) | 22 (14.4) | 18 (18) | 49 (33.5) | 9 (14.2) | 4 (4) | 27 (25.7) | 12 (32.4) | 4 (22) | 10 (12) | 223 |
