# Supplementary material for: Data on diverse roles of helix perturbations in membrane proteins
Source: Data Brief. 2016 Nov 1;9:781–802. doi: 10.1016/j.dib.2016.10.023 (PMC5099277; doi:10.1016/j.dib.2016.10.023)
Supplement: Supplementary file 2 — Supplementary material [file mmc2.zip › dib/Supplementary_Table5.docx]

**Table S5: Pair wise crossing angles for helices in the vicinity of the TM2 (reference protein)/ and structurally equivalent helix in HCO superfamily proteins.** Numbering scheme of the TM helices belongs to helices in the reference protein, corresponding helical regions have been considered from other members of the HCO family. Crossing angle values for helical regions that do not interact with the TM2/ structurally equivalent helix have been italicized and underscored.

| **Protein** | **Helices in vicinity** | | | | | |
| --- | --- | --- | --- | --- | --- | --- |
|  | **TM1** | **TM3** | **TM4** | **TM5** | **TM6** | **TM10** |
| MitochondrialCOX (1v55:A) | 155.8 | 170 | 15.1 | *48.7* | 45.9 | 37.5 |
| Ubiquinol oxidase (1fft:A) | 157 | 161 | 14.3 | *41* | 47.3 | 35 |
| Bacterial COX (3s8g:A) | 152 | *173.3* | 17.2 | 35 | 26.7 | *39* |
| Bacterial COX (1m56:A) | 154.6 | 167 | 16.3 | 30 | 44.2 | 39.4 |
| Bacterial COX (1qle:A) | 140 | 160 | 13.2 | *46* | 43.1 | 28.2 |
| Bacterial COX (3mk7:A) | 160 | 152 | 17 | 24 | *44.6* | *46.7* |
| Nitric oxide reductase (3o0r:B) | *157* | 170.3 | 13.5 | 32 | 46.2 | 48.3 |
| Nitric oxide reductase (3ayf:A) | 161 | 172 | 17.2 | 29 | 35 | 43 |
