# Supplementary material for: Data on diverse roles of helix perturbations in membrane proteins
Source: Data Brief. 2016 Nov 1;9:781–802. doi: 10.1016/j.dib.2016.10.023 (PMC5099277; doi:10.1016/j.dib.2016.10.023)
Supplement: Supplementary file 2 — Supplementary material [file mmc2.zip › dib/Supplementary_Table2.docx]

**Table 2: Main chain backbone C=O atoms which lack the helical N-H..O hydrogen bond and contribute to helical interactions in each type of perturbation.** Intra-helical interactions include the stabilization of the free backbone C=O atom by C^δ^ or C^γ^ atom of Proline and other intra-helical side chain to main chain (SM) hydrogen bonds. Inter-helical interactions include SM hydrogen bonds from amino acids belonging to the neighbouring helices and C^α^-H..O and C^β^-H..O hydrogen bonds. Numbers within parenthesis indicate percentage values.

| **Type of Perturbation** | **No. Of C=O that miss a backbone hydrogen bond** | **No. Of C=O stabilized (Intra and Inter-helical hydrogen bonds)** |
| --- | --- | --- |
| Linear-Pro | 16 | 14 (87) |
| Curved-Pro | 29 | 21 (72.4) |
| Kinked-Pro-P1 | 10 | 8 (80) |
| Kinked-Pro-P2 | 43 | 31 (72) |
| Kinked-Non-Pro | 17 | 14 (82) |
| 3_10-_ Pro | 16 | 12 (75) |
| 3_10-_ Non- Pro | 65 | 51 (78.4) |
| π-bulge-Pro | 28 | 23 (82) |
| π-bulge- Non- Pro | 14 | 12 (85) |
| Total | 218 | 186 (85.3) |
