# Supplementary material for: Data on diverse roles of helix perturbations in membrane proteins
Source: Data Brief. 2016 Nov 1;9:781–802. doi: 10.1016/j.dib.2016.10.023 (PMC5099277; doi:10.1016/j.dib.2016.10.023)
Supplement: Supplementary file 2 — Supplementary material [file mmc2.zip › dib/Supplementary_Table3.docx]

**Table 3: Proteins from the Heme-Copper Oxidase (HCO) superfamily considered for the analysis of the π-helical region.** A total of 8 proteins (at least one member of a particular HCO subtype) have been selected for analysis. The ‘Mitochondrial COX (1v55:A)’ belongs to the initial dataset of 90 proteins used for analysis and contains the interspersed 19 residue long π-helix. The ‘Helical region’ (fifth column) represents the entire TM segment considered for analysis. The ‘Helix assignment’ (sixth column) includes the helix boundaries for α and π-helices defined by ASSP (see methods).

| **Protein** | **HCO/NOR type** | **Organism** | **Resolution** | **Helical region** | **Helix assignment** |
| --- | --- | --- | --- | --- | --- |
| Mitochondrial cytochrome-c-oxidase (1v55:A) | HCO–A | *B. taurus* | 1.9 | 51-87 (37) | 51-63=α, 64-82=π, 83-87=α |
| Ubiquinol oxidase (1fft:A) | HCO–A | *E. coli* | 3.5 | 96-131 (36) | 97-110= α , 111-117= π, 118-131= α |
| Bacterial cytochrome-c-oxidase (3s8g:A) | HCO-B | *T.thermophilus* | 1.8 | 65-97 (33) | 65-71= α , 72-80=π, 81=97= α |
| Bacterial cytochrome-c-oxidase (1m56:A) | HCO-C | *R. sphaeroides* | 2.3 | 92-128 (37) | 92-104= α, 105-122=π, 123-128= α |
| Bacterial cytochrome-c-oxidase (1qle:A) | HCO-C | *P. denitrificans* | 3.0 | 84-120 (37) | 84-97= α, 98-102=π, 103-106= α, 107-115= π, 116-120= α |
| Bacterial cytochrome-c-oxidase (3mk7:A) | HCO-C | *P. stutzeri* | 3.2 | 53-85 (33) | 53-62= α, 63-69= π, 70-84= α |
| Nitric oxide reductase (3o0r:B) | cNOR | *P. aeruginosa* | 2.7 | 53-84 (32) | 53-84= α |
| Nitric oxide reductase (3ayf:A) | qNOR | *B. stearothermo*  *philus* | 2.5 | 348-379 (32) | 348-379= α |
