# Supplementary material for: Data on diverse roles of helix perturbations in membrane proteins
Source: Data Brief. 2016 Nov 1;9:781–802. doi: 10.1016/j.dib.2016.10.023 (PMC5099277; doi:10.1016/j.dib.2016.10.023)
Supplement: Supplementary file 2 — Supplementary material [file mmc2.zip › dib/Supplementary_Table4.docx]

**Table S4: Tabulated output files of ASSP and DSSP defining the long π-helical region in mitochondrial COX. ASSP defines a π-helix from (64V-82L) based on twist, rise per residue and helical radius whereas DSSP defines a π-helix from (64V-79G) denoted by the symbol ‘I’ based on backbone hydrogen bond energetics.**

**ASSP OUTPUT**

**HELIX STEP TWIST RISE VTOR BEND RADIUS**

**51 51 D 52 Q 53 I 54 Y A 101.9 1.4 48.0 94.0 2.3**

**52 52 Q 53 I 54 Y 55 N A 100.7 1.5 52.2 166.5 2.3**

**53 53 I 54 Y 55 N 56 V A 100.1 1.4 48.6 9.5 2.3**

**54 54 Y 55 N 56 V 57 V A 100.4 1.5 51.6 3.6 2.3**

**55 55 N 56 V 57 V 58 V A 100.0 1.5 51.3 3.4 2.3**

**56 56 V 57 V 58 V 59 T A 98.3 1.5 47.5 3.9 2.3**

**57 57 V 58 V 59 T 60 A A 97.3 1.5 48.6 2.5 2.3**

**58 58 V 59 T 60 A 61 H A 94.6 1.4 43.8 2.2 2.4**

**59 59 T 60 A 61 H 62 A A 103.7 1.7 60.1 7.8 2.2**

**60 60 A 61 H 62 A 63 F A 94.9 1.4 44.2 11.8 2.4**

**61 61 H 62 A 63 F 64 V A 96.2 1.5 47.6 12.0 2.3**

**62 62 A 63 F 64 V 65 M A 95.6 1.5 47.7 8.8 2.4**

**63 63 F 64 V 65 M 66 I A 94.6 1.5 45.0 3.8 2.4**

**64 64 V 65 M 66 I 67 F A 87.1 1.3 36.0 4.1 2.6**

**65 65 M 66 I 67 F 68 F A 79.7 1.0 25.1 5.8 2.8**

**66 66 I 67 F 68 F 69 M A 80.9 1.2 31.3 9.9 2.8**

**67 67 F 68 F 69 M 70 V A 74.8 1.1 25.9 4.9 3.0**

**68 68 F 69 M 70 V 71 M A 78.8 1.4 33.4 0.2 2.8**

**69 69 M 70 V 71 M 72 P A 89.2 1.5 41.6 6.1 2.5**

**70 70 V 71 M 72 P 73 I A 96.6 1.5 49.0 4.1 2.3**

**71 71 M 72 P 73 I 74 M A 92.7 1.5 44.1 4.4 2.4**

**72 72 P 73 I 74 M 75 I A 86.1 1.4 37.0 4.9 2.6**

**73 73 I 74 M 75 I 76 G A 79.5 0.8 21.1 9.2 2.9**

**74 74 M 75 I 76 G 77 G A 85.6 1.3 34.5 14.4 2.6**

**75 75 I 76 G 77 G 78 F A 82.8 1.2 32.5 12.2 2.7**

**76 76 G 77 G 78 F 79 G A 81.8 0.9 23.5 15.5 2.8**

**77 77 G 78 F 79 G 80 N A 99.7 1.7 55.5 19.8 2.2**

**78 78 F 79 G 80 N 81 W A 92.3 1.3 40.4 19.5 2.5**

**79 79 G 80 N 81 W 82 L A 93.7 1.5 45.2 19.3 2.4**

**80 80 N 81 W 82 L 83 V A 78.3 1.1 26.5 4.3 2.9**

**81 81 W 82 L 83 V 84 P A 97.7 2.2 67.3 20.8 2.0**

**82 82 L 83 V 84 P 85 L A 93.9 1.4 43.0 27.2 2.4**

**83 83 V 84 P 85 L 86 M A 98.7 1.5 48.8 31.9 2.3**

**84 84 P 85 L 86 M 87 I A 99.6 1.7 55.7 25.6 2.2**

**85 85 L 86 M 87 I 88 G A 91.3 1.2 36.6 15.0 2.5**

**86 86 M 87 I 88 G 89 A A 227.9 2.6 247.0 94.2 1.5**

**DSSP OUTPUT**

**RESIDUE AA STRUCTURE BP1 BP2 ACC N-H-->O O-->H-N N-H-->O O-->H-N**

**51 51 A D H 3> S+ 0 0 74 -2,-0.3 4,-2.3 1,-0.2 5,-0.1
 52 52 A Q H 3> S+ 0 0 58 2,-0.2 4,-1.9 1,-0.2 -1,-0.2
 53 53 A I H <> S+ 0 0 76 -3,-0.5 4,-2.3 2,-0.2 -2,-0.2
 54 54 A Y H X S+ 0 0 2 -4,-1.8 4,-2.3 1,-0.2 -2,-0.2
 55 55 A N H X S+ 0 0 49 -4,-2.3 4,-2.2 1,-0.2 70,-0.4
 56 56 A V H X S+ 0 0 18 -4,-1.9 4,-2.7 67,-0.2 -1,-0.2
 57 57 A V H X S+ 0 0 15 -4,-2.3 4,-2.3 2,-0.2 -2,-0.2
 58 58 A V H X S+ 0 0 27 -4,-2.3 4,-1.8 2,-0.2 -2,-0.2
 59 59 A T H X S+ 0 0 4 -4,-2.2 4,-2.0 1,-0.2 -2,-0.2
 60 60 A A H X S+ 0 0 13 -4,-2.7 4,-2.8 1,-0.2 5,-0.3
 61 61 A H H X S+ 0 0 24 -4,-2.3 4,-2.4 -5,-0.2 -1,-0.2
 62 62 A A H X S+ 0 0 17 -4,-1.8 4,-2.4 2,-0.2 5,-0.3
 63 63 A F H X>S+ 0 0 5 -4,-2.0 4,-2.2 1,-0.2 5,-0.8
 64 64 A V I X>S+ 0 0 0 -4,-2.8 5,-1.5 3,-0.2 4,-1.4
 65 65 A M I <>S+ 0 0 40 -4,-2.4 5,-2.9 -5,-0.3 -2,-0.2
 66 66 A I I <>S+ 0 0 14 -4,-2.4 5,-2.0 -5,-0.2 -2,-0.2
 67 67 A F I <5S+ 0 0 15 -4,-2.2 -3,-0.2 -5,-0.3 -2,-0.1
 68 68 A F I <<S+ 0 0 2 -4,-1.4 -42,-0.3 -5,-0.8 -41,-0.2
 69 69 A M I ><S+ 0 0 15 -5,-1.5 4,-2.0 -6,-0.2 5,-0.2
 70 70 A V I ><S+ 0 0 8 -5,-2.9 4,-2.6 1,-0.2 5,-0.3
 71 71 A M H >XS+ 0 0 7 -5,-2.0 4,-1.9 1,-0.2 5,-1.3
 72 72 A P I 4>S+ 0 0 3 0, 0.0 5,-1.7 0, 0.0 -1,-0.2
 73 73 A I I <>S+ 0 0 14 -4,-2.0 5,-2.7 3,-0.2 6,-0.3
 74 74 A M I <>S+ 0 0 2 -4,-2.6 5,-1.0 -5,-0.2 -3,-0.2
 75 75 A I I <>S+ 0 0 17 -4,-1.9 5,-0.7 -5,-0.3 -2,-0.1
 76 76 A G I <S+ 0 0 3 -5,-1.3 4,-0.4 3,-0.2 -57,-0.2
 77 77 A G I ><S+ 0 0 0 -5,-1.7 4,-2.3 -6,-0.3 5,-0.2
 78 78 A F I ><S+ 0 0 0 -5,-2.7 4,-2.7 1,-0.2 5,-0.4
 79 79 A G I ><S+ 0 0 0 -5,-1.0 4,-2.3 -6,-0.3 -3,-0.2
 80 80 A N H 4<S+ 0 0 0 -5,-0.7 5,-0.2 -4,-0.4 -2,-0.2
 81 81 A W H X S+ 0 0 8 -4,-2.3 4,-0.6 -5,-0.1 -2,-0.2
 82 82 A L H X S+ 0 0 0 -4,-2.7 4,-2.8 -5,-0.2 -3,-0.2
 83 83 A V H X S+ 0 0 0 -4,-2.3 4,-1.0 -5,-0.4 6,-0.2
 84 84 A P H >4>S+ 0 0 0 0, 0.0 5,-2.6 0, 0.0 3,-0.7
 85 85 A L H ><5S+ 0 0 1 -4,-0.6 3,-0.9 1,-0.2 -2,-0.2
 86 86 A M H 3<5S+ 0 0 4 -4,-2.8 -1,-0.2 1,-0.2 406,-0.2
 87 87 A I T <<5S- 0 0 1 -4,-1.0 -1,-0.2 -3,-0.7 -2,-0.2**
