# Supplementary figures and images for: Data on diverse roles of helix perturbations in membrane proteins
Source: Data Brief. 2016 Nov 1;9:781–802. doi: 10.1016/j.dib.2016.10.023 (PMC5099277; doi:10.1016/j.dib.2016.10.023)

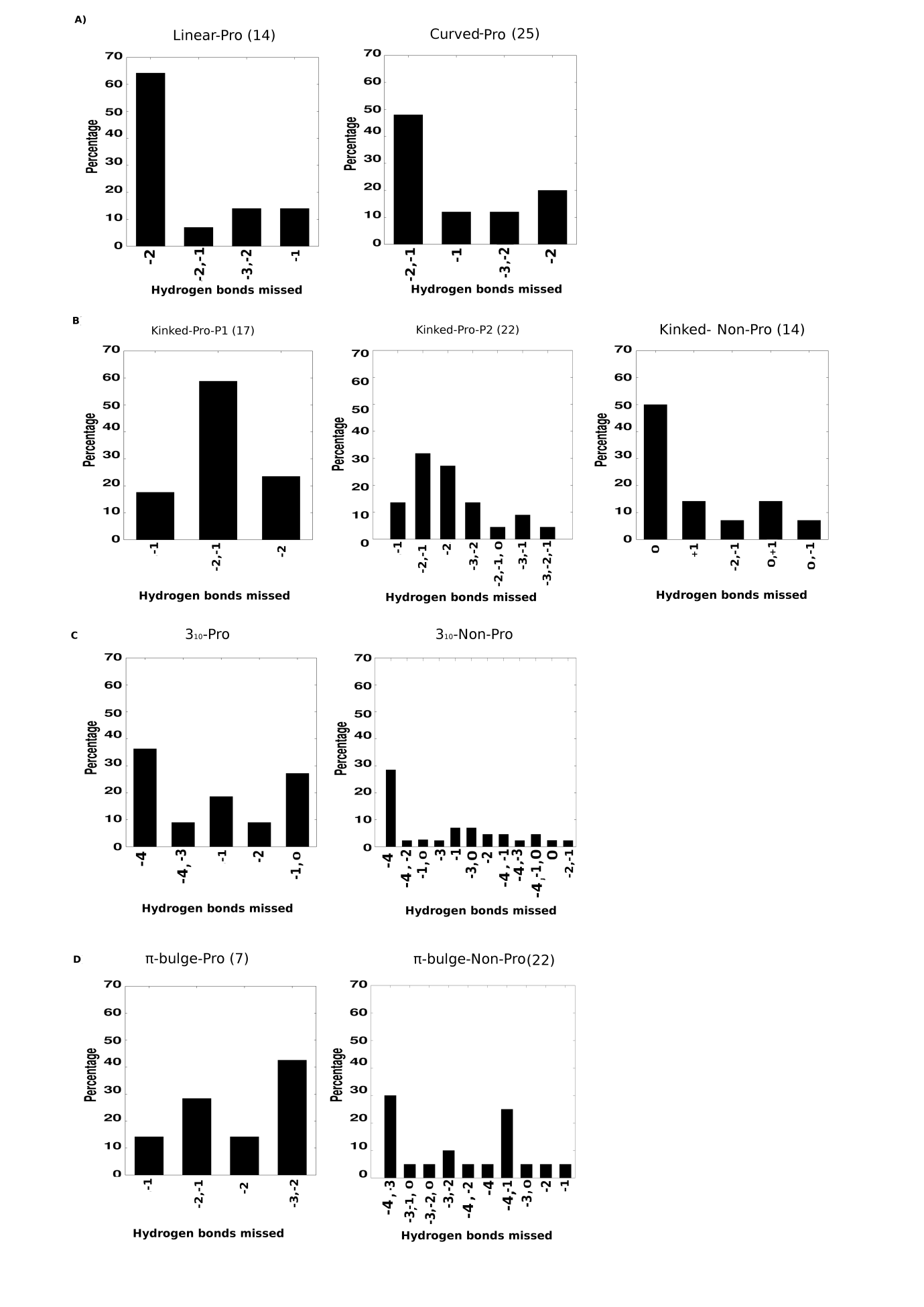

Supplement: Supplementary file 2 — Supplementary material [file mmc2.zip › dib/Supplementary_Figure8.png]

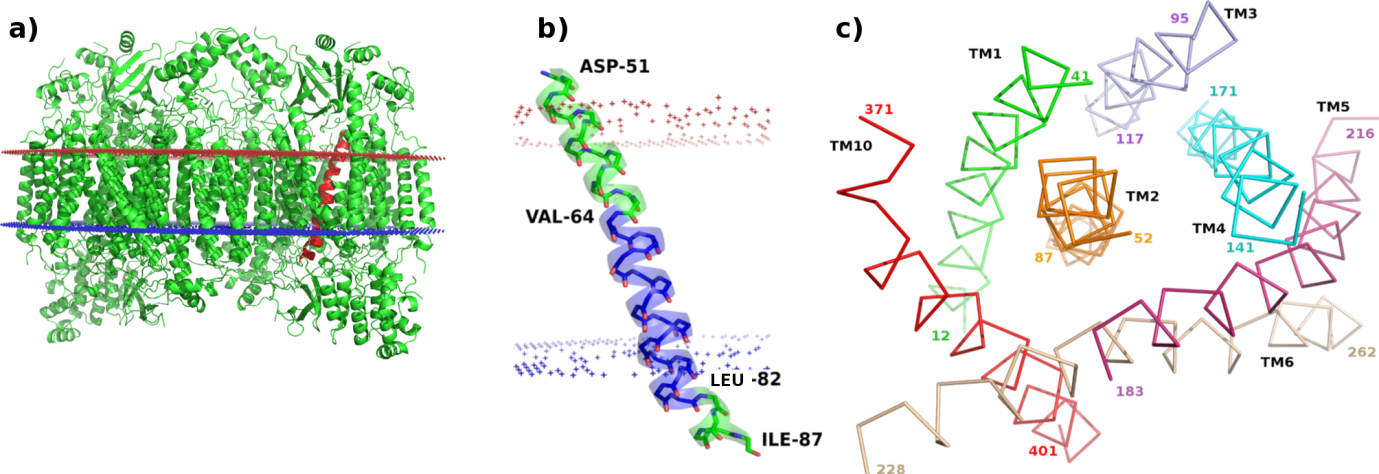

Supplement: Supplementary file 2 — Supplementary material [file mmc2.zip › dib/Supplementary_Figure14.png]

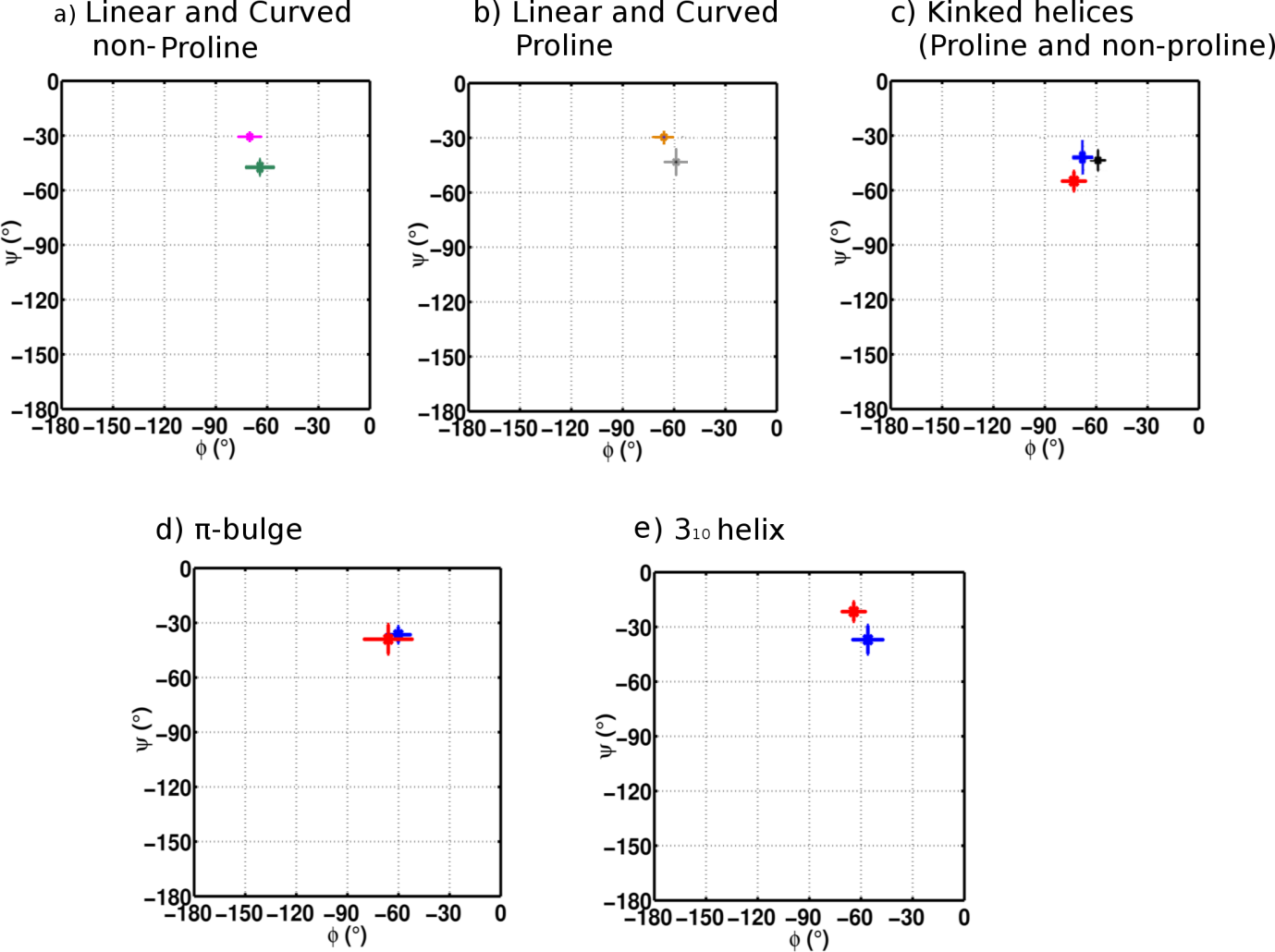

Supplement: Supplementary file 2 — Supplementary material [file mmc2.zip › dib/Supplementary_Figure9.png]

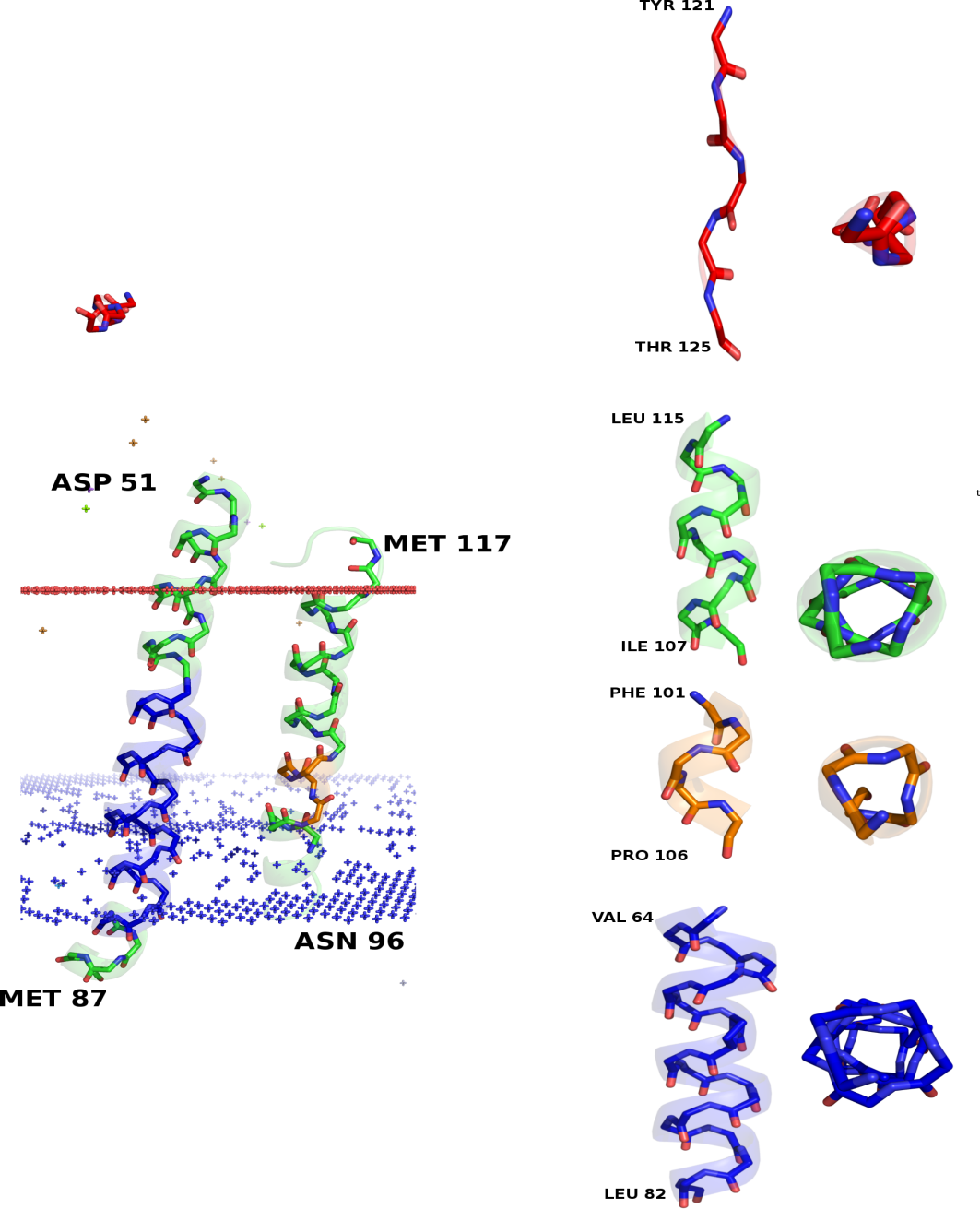

Supplement: Supplementary file 2 — Supplementary material [file mmc2.zip › dib/Supplementary_Figure2.png]

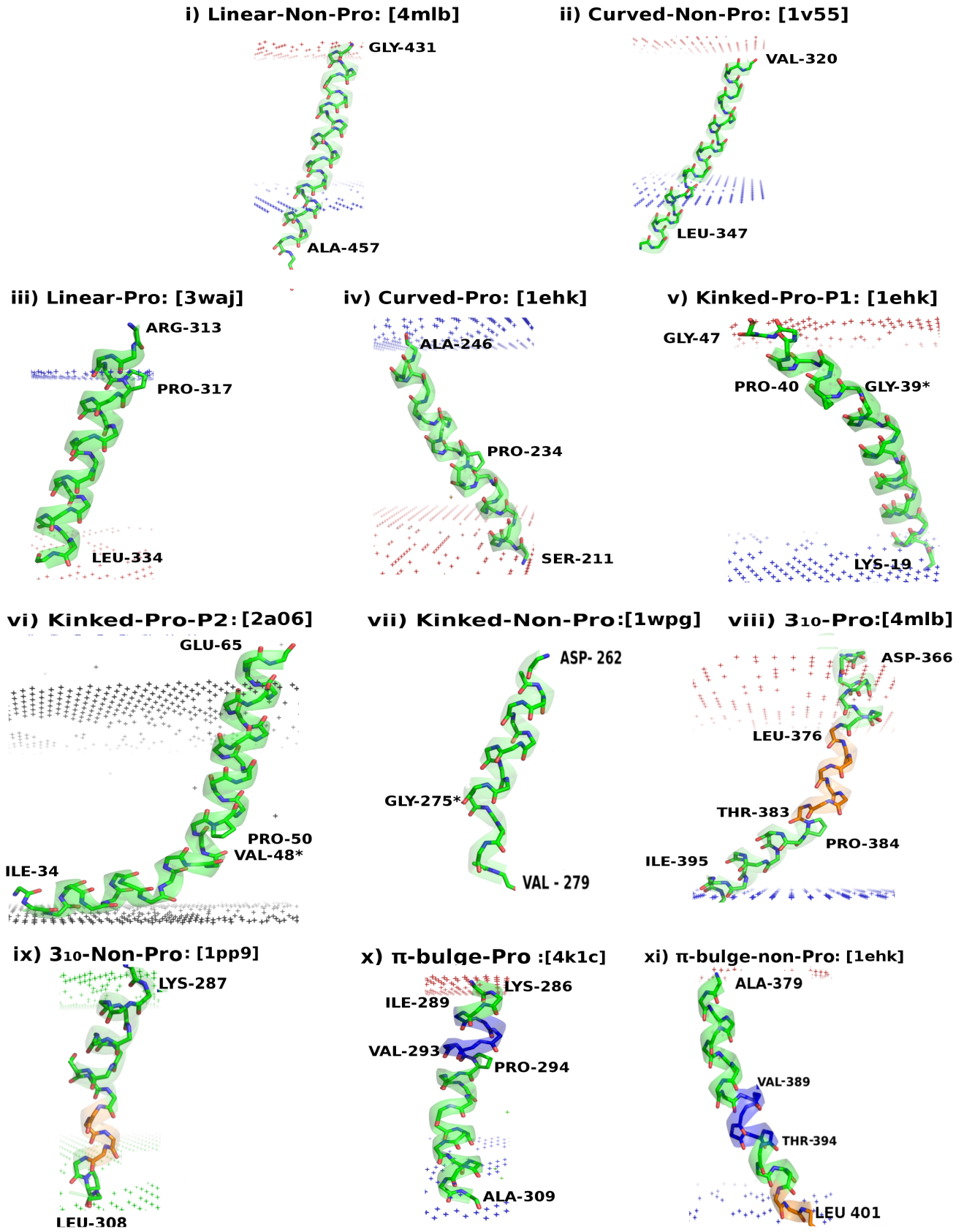

Supplement: Supplementary file 2 — Supplementary material [file mmc2.zip › dib/Supplementary_Figure4.png]

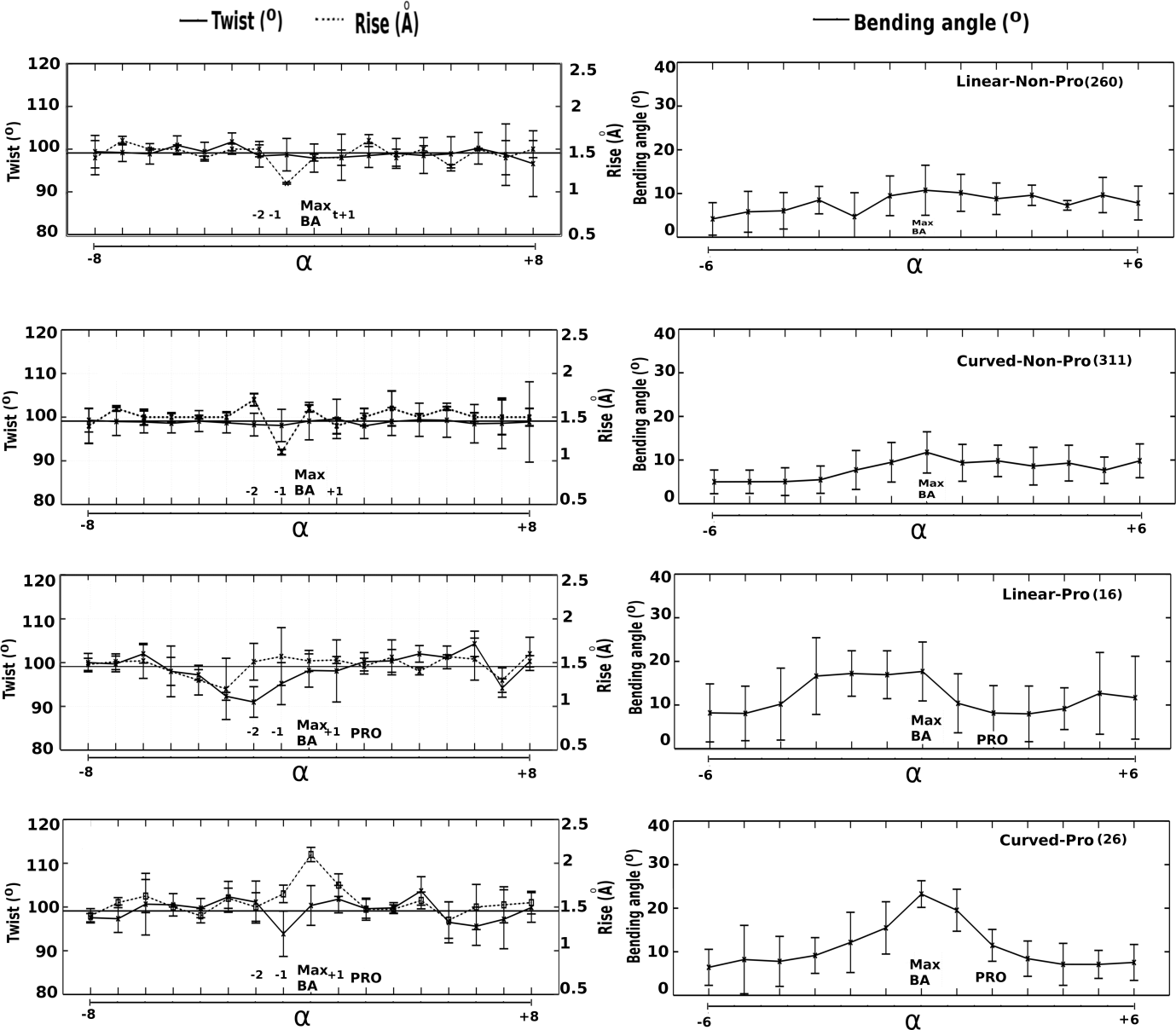

Supplement: Supplementary file 2 — Supplementary material [file mmc2.zip › dib/Supplementary_Figure5.png]

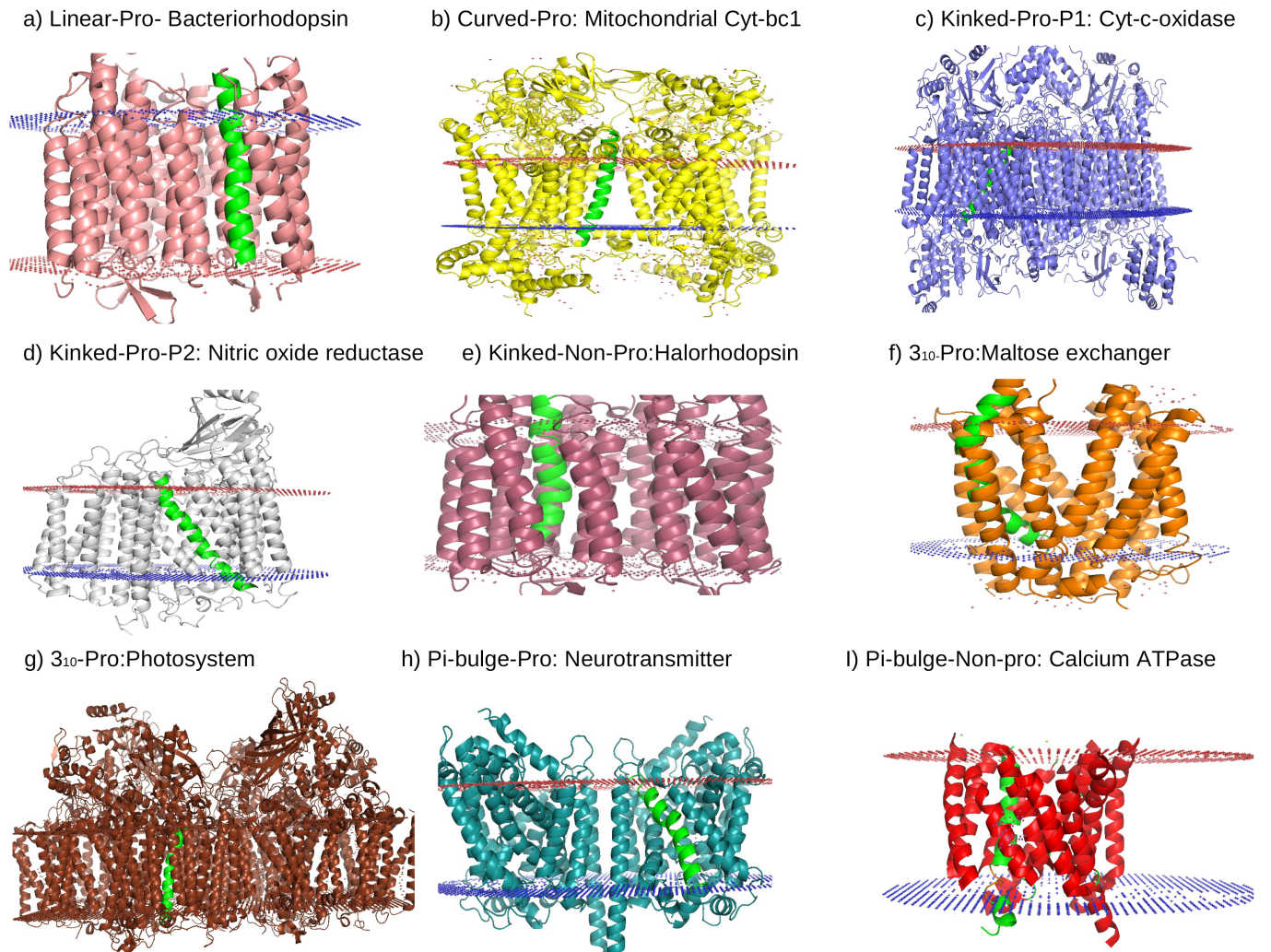

Supplement: Supplementary file 2 — Supplementary material [file mmc2.zip › dib/Supplementary_Figure12.png]

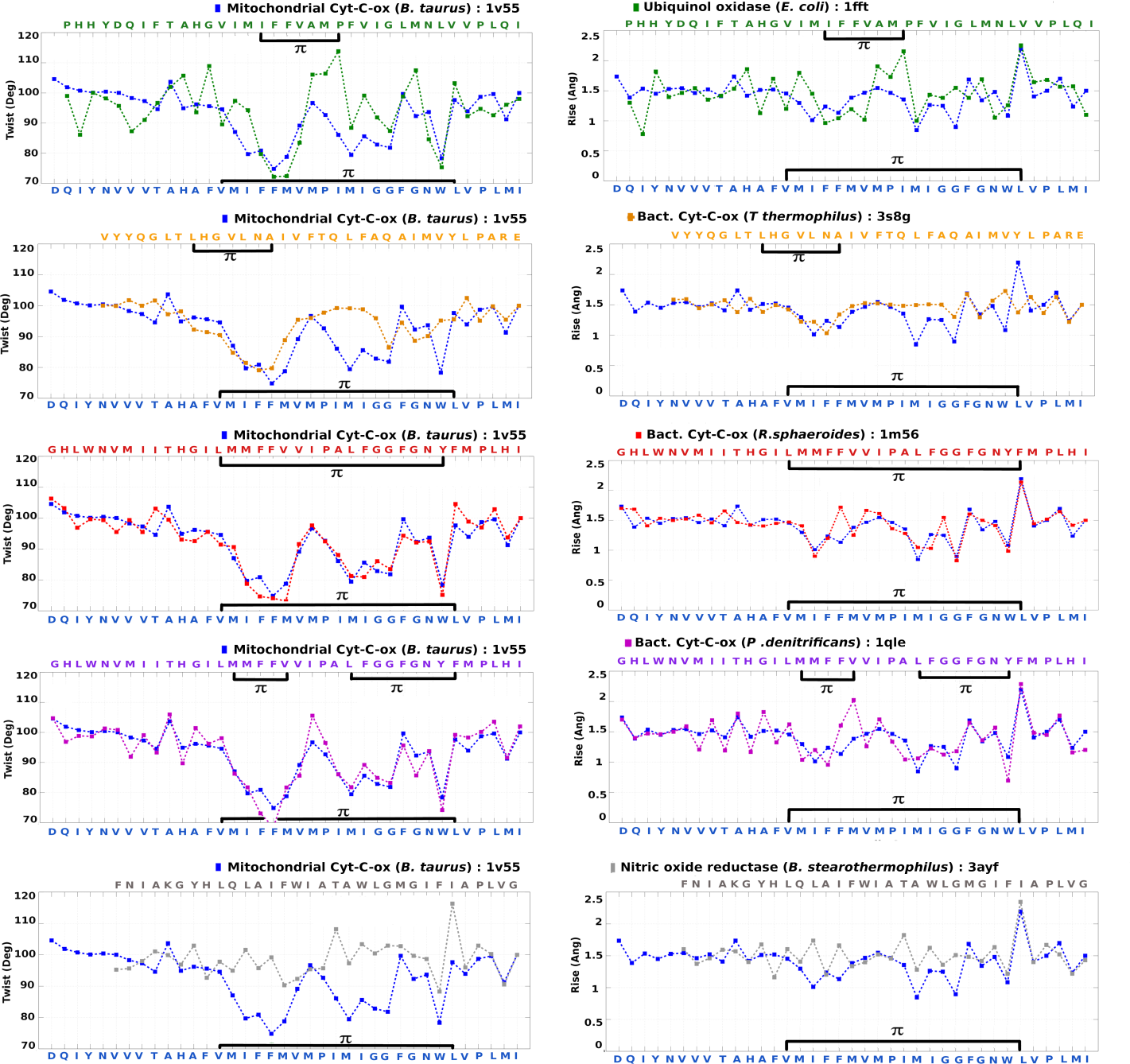

Supplement: Supplementary file 2 — Supplementary material [file mmc2.zip › dib/Supplementary_Figure16.png]

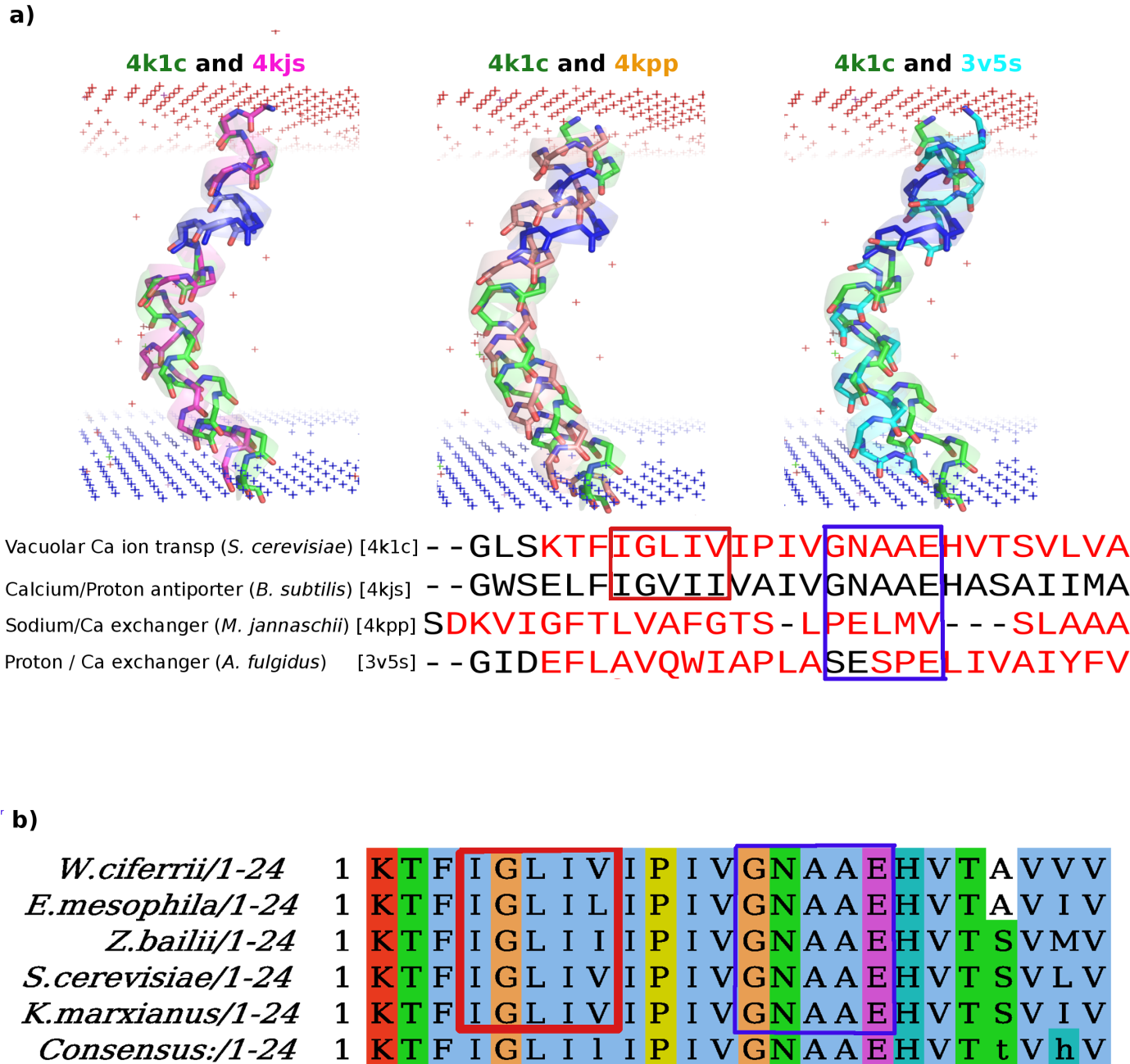

Supplement: Supplementary file 2 — Supplementary material [file mmc2.zip › dib/Supplementary_Figure13.png]

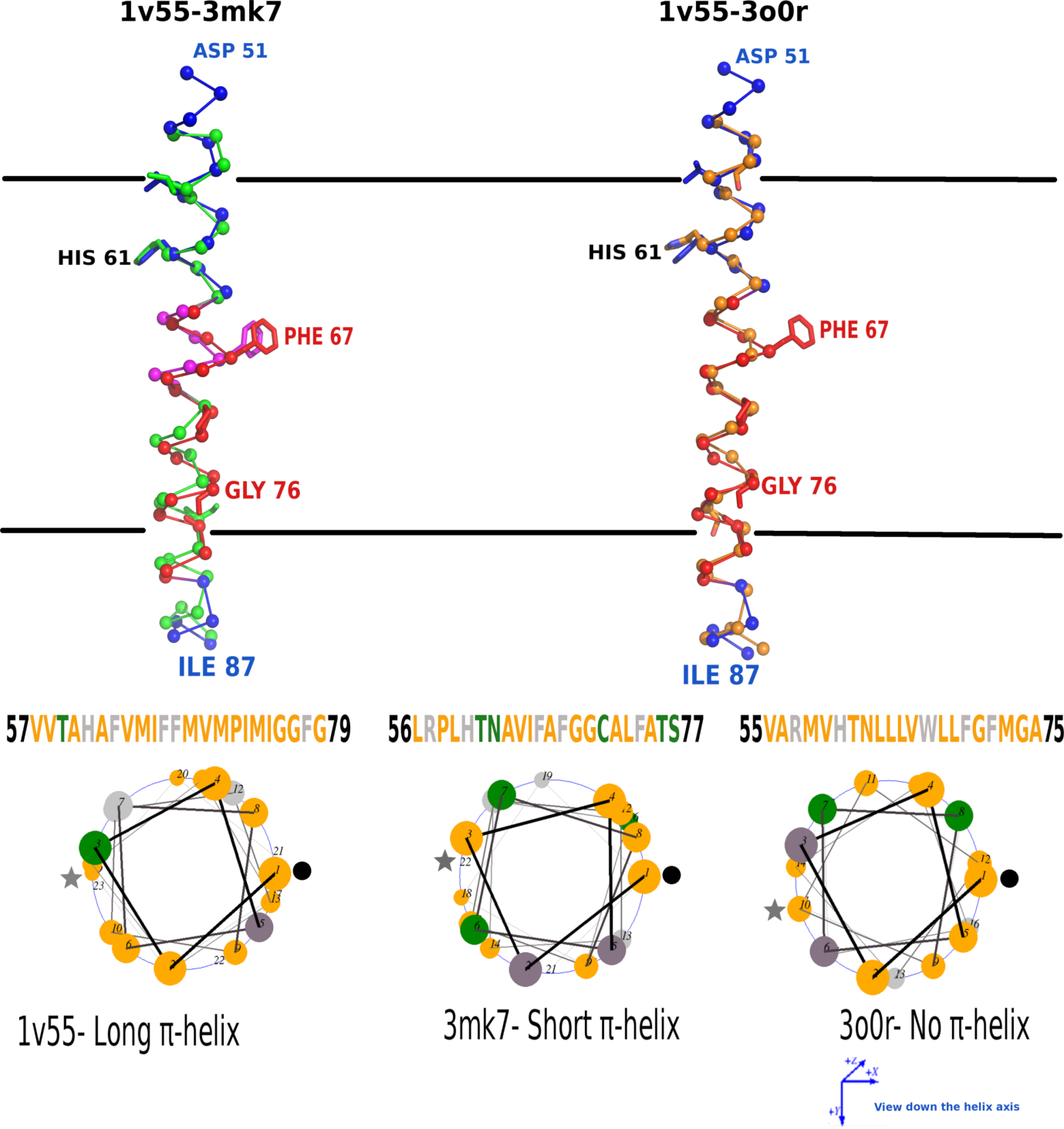

Supplement: Supplementary file 2 — Supplementary material [file mmc2.zip › dib/Supplementary_Figure17.png]

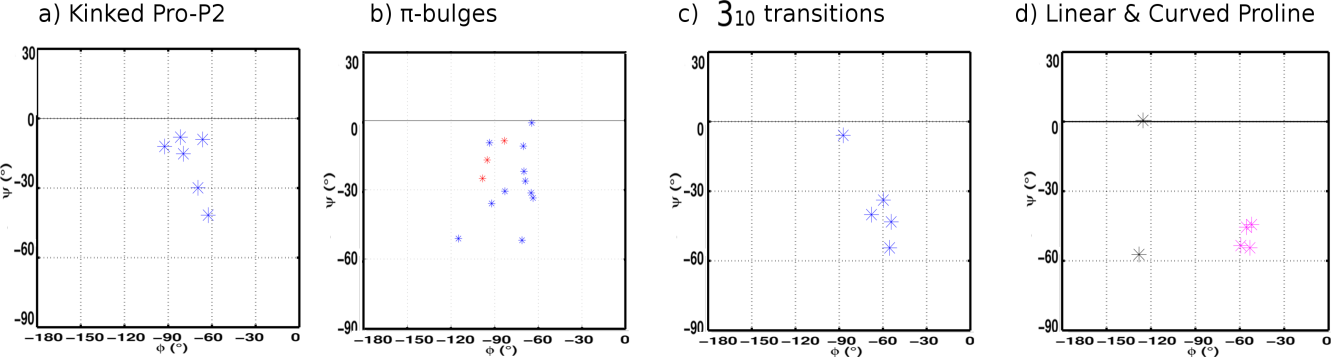

Supplement: Supplementary file 2 — Supplementary material [file mmc2.zip › dib/Supplementary_Figure7.png]

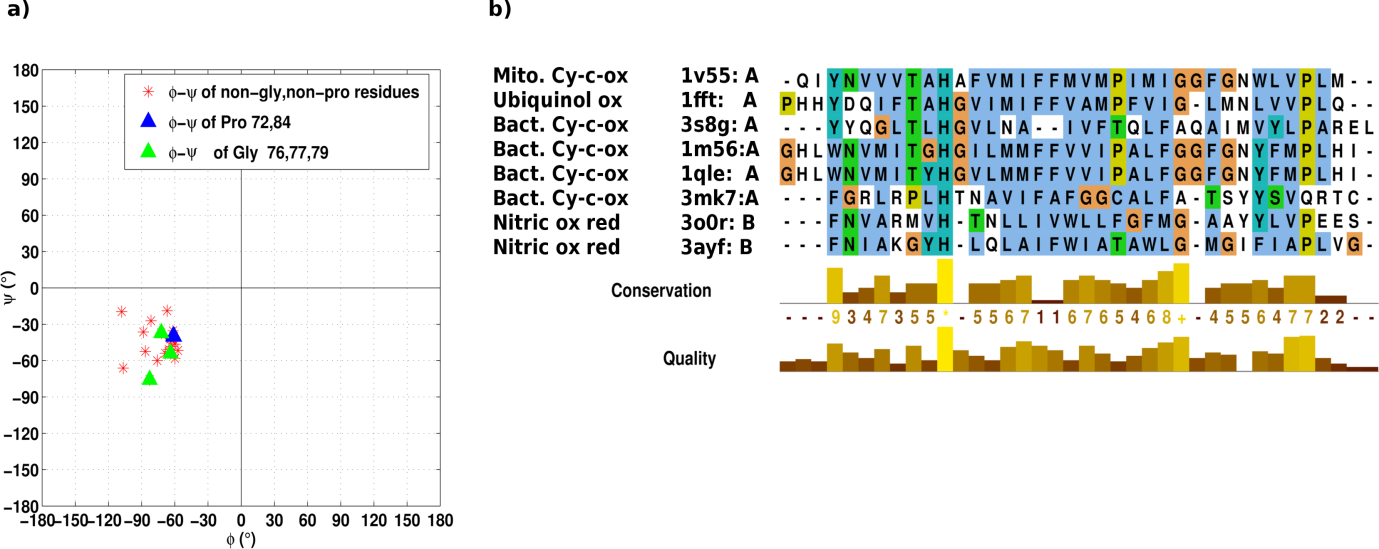

Supplement: Supplementary file 2 — Supplementary material [file mmc2.zip › dib/Supplementary_Figure15.png]

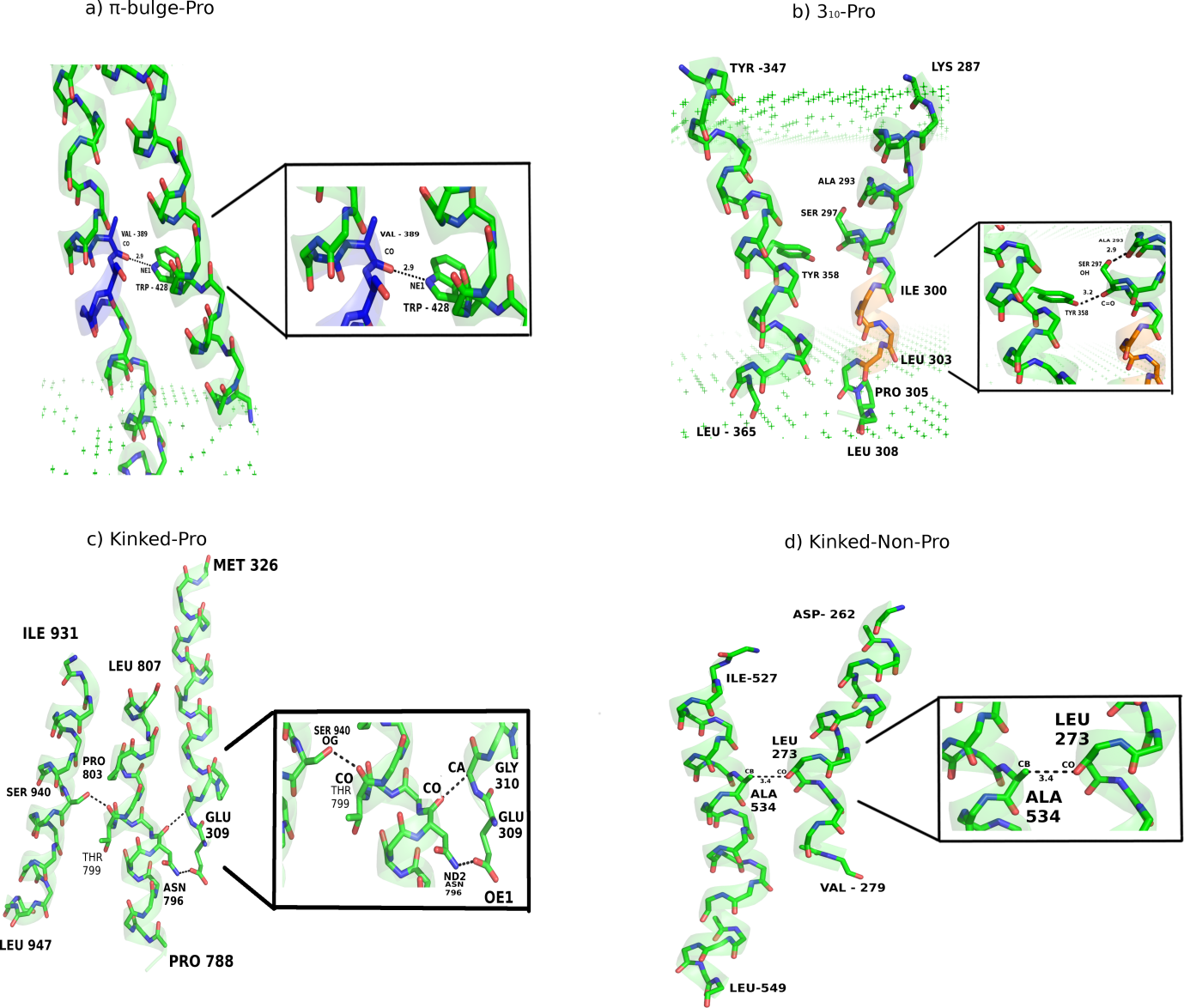

Supplement: Supplementary file 2 — Supplementary material [file mmc2.zip › dib/Supplementary_Figure10.png]

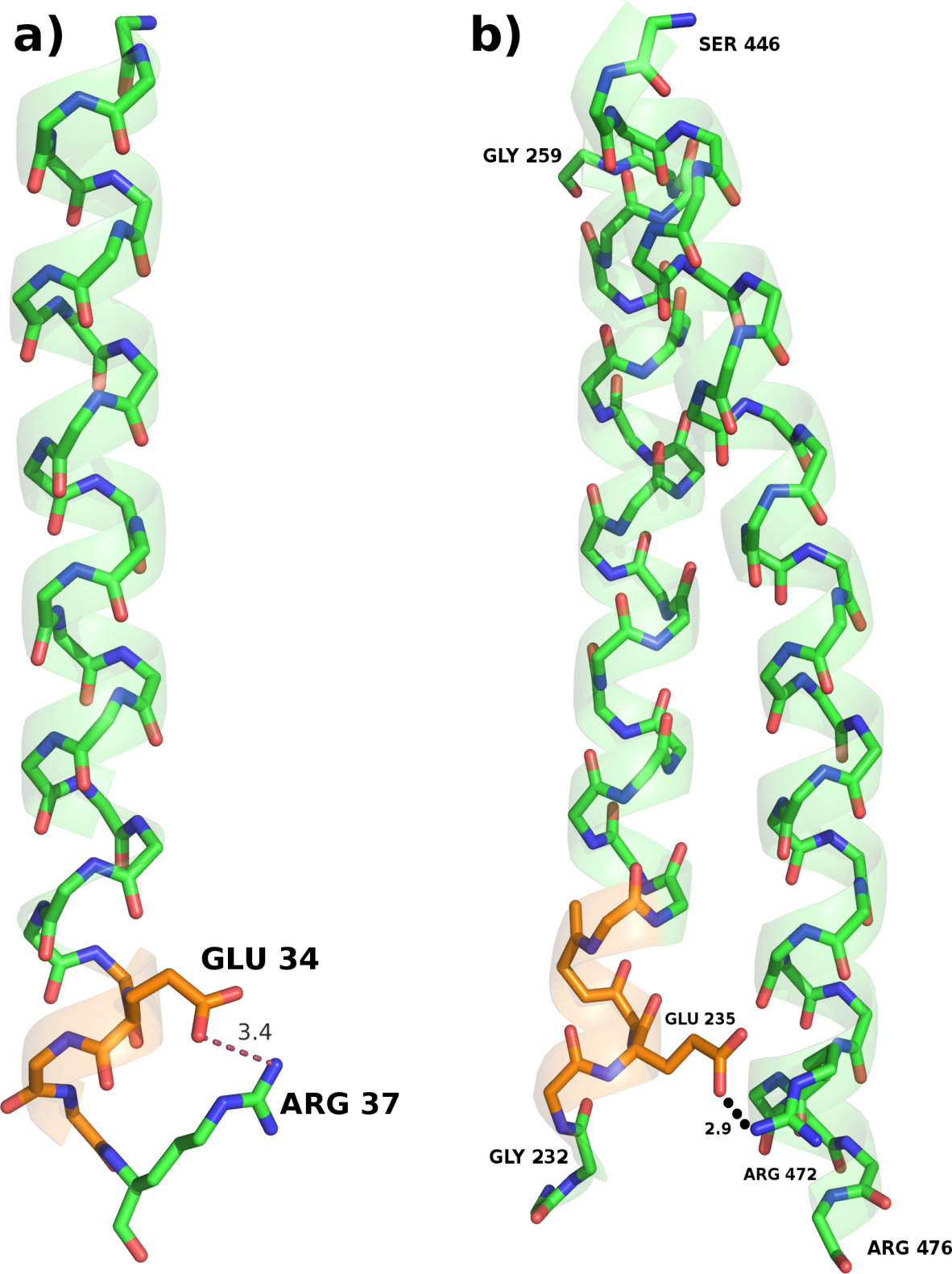

Supplement: Supplementary file 2 — Supplementary material [file mmc2.zip › dib/Supplementary_Figure3.png]

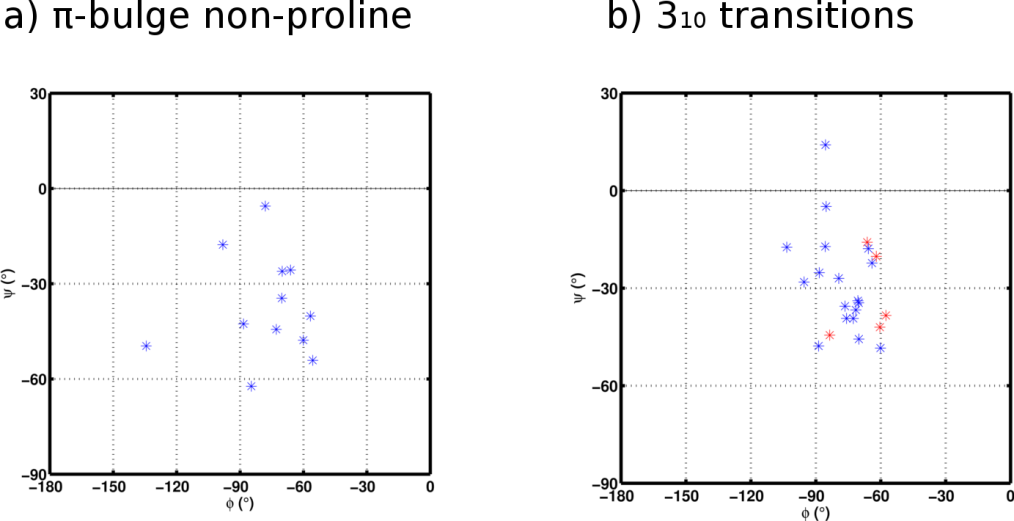

Supplement: Supplementary file 2 — Supplementary material [file mmc2.zip › dib/Supplementary_Figure6.png]

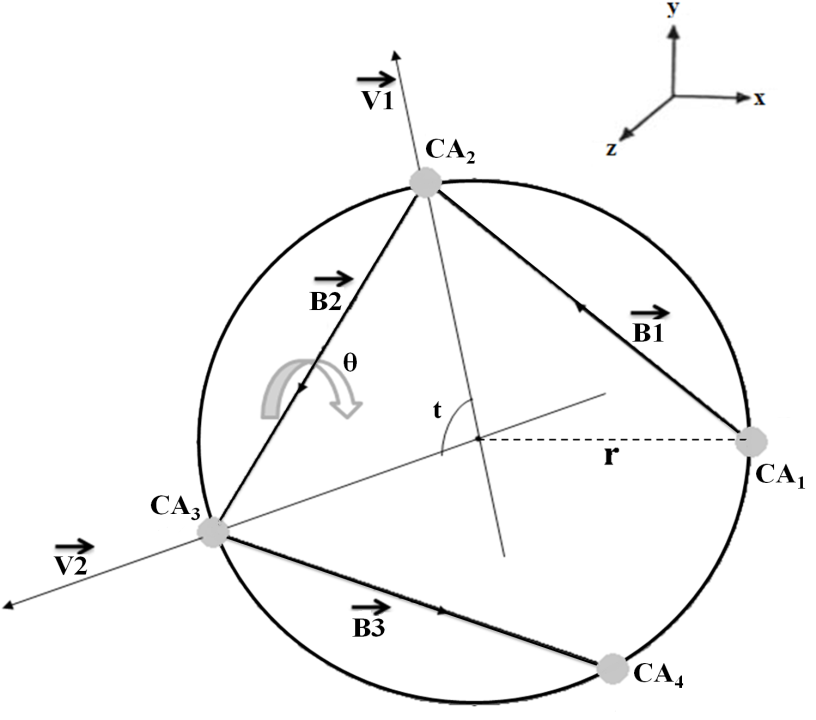

Supplement: Supplementary file 2 — Supplementary material [file mmc2.zip › dib/Supplementary_Figure1.png]

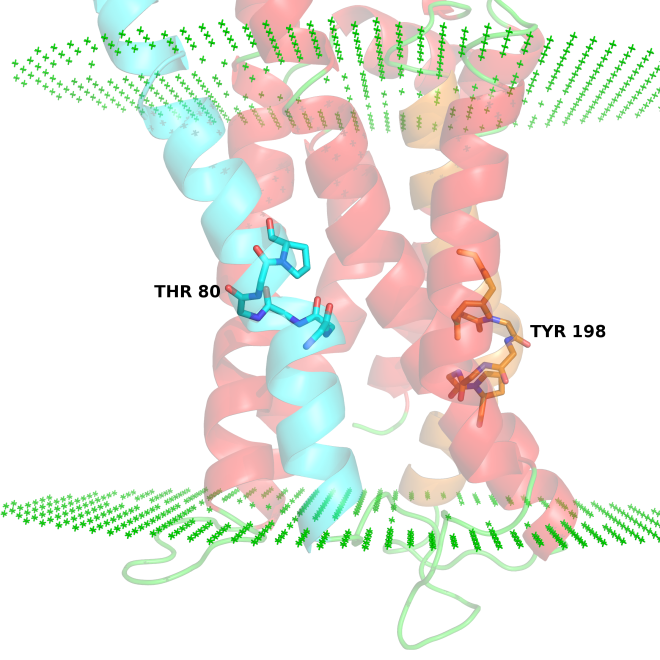

Supplement: Supplementary file 2 — Supplementary material [file mmc2.zip › dib/Supplementary_Figure11.png]
